# Atypical structures of GAA/TTC trinucleotide repeats underlying Friedreich’s ataxia: DNA triplexes and RNA/DNA hybrids

**DOI:** 10.1093/nar/gkaa665

**Published:** 2020-08-21

**Authors:** Jiahui Zhang, Ashkan Fakharzadeh, Feng Pan, Christopher Roland, Celeste Sagui

**Affiliations:** Department of Physics, North Carolina State University, Raleigh, NC 27695-8202, USA; Department of Physics, North Carolina State University, Raleigh, NC 27695-8202, USA; Department of Physics, North Carolina State University, Raleigh, NC 27695-8202, USA; Department of Statistics, Florida State University, Tallahassee, FL 32306, USA; Department of Physics, North Carolina State University, Raleigh, NC 27695-8202, USA; Department of Physics, North Carolina State University, Raleigh, NC 27695-8202, USA

## Abstract

Expansion of the GAA/TTC repeats in the first intron of the FXN gene causes Friedreich’s ataxia. Non-canonical structures are linked to this expansion. DNA triplexes and R-loops are believed to arrest transcription, which results in frataxin deficiency and eventual neurodegeneration. We present a systematic *in silico* characterization of the possible DNA triplexes that could be assembled with GAA and TTC strands; the two hybrid duplexes [r(GAA):d(TTC) and d(GAA):r(UUC)] in an R-loop; and three hybrid triplexes that could form during bidirectional transcription when the non-template DNA strand bonds with the hybrid duplex (collapsed R-loops, where the two DNA strands remain antiparallel). For both Y·R:Y and R·R:Y DNA triplexes, the parallel third strand orientation is more stable; both parallel and antiparallel protonated d(GA^+^A)·d(GAA):d(TTC) triplexes are stable. Apparent contradictions in the literature about the R·R:Y triplex stability is probably due to lack of molecular resolution, since shifting the third strand by a single nucleotide alters the stability ranking. In the collapsed R-loops, antiparallel d(TTC^+^)·d(GAA):r(UUC) is unstable, while parallel d(GAA)·r(GAA):d(TTC) and d(GA^+^A)·r(GAA):d(TTC) are stable. In addition to providing new structural perspectives for specific therapeutic aims, our results contribute to a systematic structural basis for the emerging field of quantitative R-loop biology.

## INTRODUCTION

Trinucleotide repeats (TRs) exhibit ‘dynamic mutations’ that cause them to expand. After crossing a critical threshold length, the expansion gives rise to trinucleotide repeat expansion disorders (TREDs) (1–7). These are inherited neurological disorders that exhibit a phenomenon known as ‘anticipation’, where the age of the onset of the disease typically decreases and the severity of the disease phenotype typically increases in each subsequent generation (4,8–11). Longer repeat tracts become progressively more deleterious and constitute the major molecular determinant of anticipation in a significant number of diseases, with other genetic modifiers and environmental factors accounting for the remainder of the effect (11,12). The expansion of microsatellite repeats is behind 50 neurodegenerative and neuromuscular disorders (11,13–17). The repetitive, sequential structure of TRs causes slippage during DNA replication, repair, transcription and/or recombination (10–14,18–22) leading to expansion and high mutation rates.

Although the mechanisms underlying TREDs are understood to be extremely complex, an important breakthrough has been the recognition that the critical step in all models of repeat instability is the transient formation of atypical, non-B DNA stable secondary structures in the expandable repeats (17,18,23–25). Indeed, expandable repeats have been shown to display atypical structural characteristics, including single-stranded hairpins, Z-DNA, triple helices, G-quartets, slipped-stranded duplexes and R-loops. Interestingly, the quest to understand the molecular mechanisms behind these diseases has also opened a window into the understanding of the less known atypical secondary structures of nucleic acids, thus contributing to the basic field of nucleic acids research.

In this work, we present results related to the atypical structures associated with GAA/TTC TRs. Friedreich’s ataxia (FRDA) is caused by a GAA expansion in the first intron of the frataxin (FXN) gene (the convention is that the repeat on the coding strand is considered the disease-causing repeat for a specific locus). Experimentally, GAA/TTC repeats have been found to form either triplexes or R-loops. A DNA triplex (also known as H-DNA or triple-stranded DNA) was first reported in 1957 (26). This is a non-canonical three-stranded structure consisting of a Watson–Crick paired helical duplex and a third strand that binds to this duplex via Hoogsteen or reversed Hoogsteen hydrogen bonds. In the most general case, DNA triplexes can occur in the cell naturally in the form of intramolecular triplexes formed at endogenous mirror repeats of oligopyrimidine/oligopurine sequences or by applying an exogenous oligonucleotide, generally known as TFO—triplex-forming oligonucleotide (27,28). In either case, these triplexes can induce transcriptional repression and site-specific mutagenesis or recombination (27). In particular, TFOs can bind to sequence specific DNA duplexes and thus present enormous gene therapeutical potential (28). GAA/TTC sequences, representing pure purine and pure pyrimidine strands, are thus excellent candidates for triplex formation. Using the standard notation (29) where R represents a pure purine strand (not to be confused with the ‘R’ in an ‘R-loop’) and Y represents a pure pyrimidine strand, such that R:Y is the usual B-DNA duplex, then both R·R:Y (purine third strand) and Y·R:Y (pyrimidine third strand) triplexes have been found in GAA/TTC sequences (30–38).

An R-loop is a three-stranded nucleic acid structures consisting of a hybrid RNA:DNA duplex formed by the template DNA and the RNA strands, along with the displaced, non-template, single-strand DNA. They were initially observed in DNA replication, and are also observed during transcription. R-loops can regulate cellular processes such as gene expression, DNA replication and repair, and immunoglobulin class-switch recombination, and as such they have been intensively studied (39,40). They can also cause DNA damage and genome instability, and recently, they have been linked to neurodegenerative diseases such as Friedreich’s Ataxia (41). While all sequences make temporary R-loops behind RNA polymerase II (RNAPII), loops created in regions with asymmetric purine-rich and pyrimidine-rich strands are susceptible to form stable, long-lived hybrids (42). The all-purine GAA and all-pyrimidine TTC strands are perfect candidates for R-loops (41,43,44), and the hybrid duplexes can form in either of the two DNA strands due to bidirectional transcription (45–47). In particular, increased expression of the FXN Antisense Transcript 1 (FAST-1) has been shown to cause heterochromatin formation and transcriptional silencing of the FXN gene (48). An intriguing possibility is the formation of a hybrid triple helix, where the ssDNA left behind by RNAPII is no longer ‘loose’ but attaches through Hoogsteen or reversed Hoogsteen hydrogen bonds to the hybrid DNA:RNA helix, thus forming a hybrid triplex (or ‘collapsed R-loop’ (49)).

In this work, we report simulation results about atypical secondary structures associated with GAA/TTC TRs, obtained via classical *atomistic* molecular dynamics (MD) simulations. Atomistic MD tools have proved extremely valuable as they possess the ability to determine molecular structures, dynamics and mechanisms at the atomic level, which are often beyond the resolution of experiments. Our recent characterization of homoduplexes, quadruplexes and hairpins conformations corresponding to the most common TRs and to several hexanucleotide repeats is an example of that (50–56), as these simulations sample both DNA and RNA sequences of different lengths, different non-equivalent nucleotide arrangements (such as (GCC)_*n*_ and (CCG)_*n*_ homoduplexes, with CpG and GpC steps between the C–C mismatches); provide free energies, dynamics of conformational transitions etc. These conformation studies do not address the formation of the atypical structures: In the cell, in order for these atypical structures to be formed, it is necessary to cross a free energy barrier that is sequence and repeat-length dependent. One of the advantages of MD is that simulations can start in any minimum of the free energy. Thus, one can simply study the resulting structure after its nucleation has taken place (similarly to studying a folded protein after folding has taken place), and the length-dependence for the nucleation becomes irrelevant. In this sense, MD studies are the same as the (relatively scarce) X-ray and NMR studies (generally with very few repeats) that report on the already formed atypical structures of TRs. In this work, we explore the structure and stability of DNA triplexes by studying 16 geometrically different GAA/TTC triplexes; we present results with respect to the two hybrid RNA:DNA helical duplexes; and results related to the three possible DNA·DNA:RNA hybrid triplexes that can form a collapsed R-loop. Even though the formation and stability of DNA triplexes has been reported experimentally (30–38), a search of the Protein Data Bank reveals no molecular structure for the GAA/TTC triplexes. Our work provides a systematic characterization of these structures and their relative stability, the particular hydrogen bond patterns and the symmetry correlation between various triplexes. In particular, we show that apparent contradictions in the literature about the stability of R·R:Y triplexes is probably due to lack of molecular resolution, since shifting the third strand by a single nucleotide alters the stability ranking. We also probe collapsed R-loops, that have not been studied before, and rate their stability. Our work contributes to the understanding of the atypical structures related to GAA/TTC expansions. In addition, the definition of R-loops has recently been extended to include a large class of non-canonical structures suggesting a novel level of biological complexity (57). There is, however, scarce experimental data with molecular resolution for these structures. Our work contributes to widen the structural knowledge of the R-loop repertoire. The fact that several of the structures presented here are stable strongly suggests that they may either coexist or compete in the transcriptional R-loop.

## MATERIALS AND METHODS

There are two parts to the work presented here: the initial construction of the atypical secondary structures and the subsequent MD simulations based on the initial model triple helices. The initial structures for the triple helices were constructed *de novo* using single strands with three repeats (nine nucleotides) each; the process involves both the careful determination of the relevant hydrogen bonds between the third strand and the canonical duplex, and the assembly of the three strands. The modeling process and the resulting initial structures are described in the next section.

The MD simulations were carried out with the Amber18 package (58) with force field BSC1 (59) (DNA part) and BSC0 (60) + OL3 (61) (RNA part) for different atypical structures combined with the protonated AMBER force field (62). The protonation was completed with tleap (58). The simulations for the pyrimidine third strand were carried both with and without protonation for the cytosine. The TIP3P water model (63) was used for the explicit solvent simulations under periodic boundary conditions in truncated octahedron water boxes. The appropriate number of Na^+^ ions (parameters in (64)) was used for neutralization of the nucleic acid charges. Additional simulations were run with Mg^2 +^ ions (parameters in (65)) in a 0.20M concentration, with Cl^−^ ions added for neutralization. One run, apR was additionally run with 40 and 80 mM concentrations.

Electrostatics were handled by the Particle Mesh Ewald method (66), with a direct space cutoff of 9 Å. The cutoff for van der Waals interactions was set as 9 Å. We used Langevin dynamics with a coupling parameter 1.0 ps^−1^. The SHAKE algorithm (67) was applied to all bonds involving hydrogen atoms. Hydrogen bonds were identified by cpptraj as supplied by ambertools18 (58) with a distance cutoff of 3.5 Å and an angle cutoff of 140°.

Starting conformations for MD calculations were obtained as follows. We first minimized the energy for the initial conformations obtained by modeling: first, by keeping the nucleic acid and ions fixed; then, by allowing them to move. Subsequently, the temperature was gradually raised using constant volume simulations from 0 to 300 K over 50 ps runs with a 1 fs time step. Then a 100 ps run at constant volume was used to gradually reduce the restraining harmonic constants for nucleic acids and ions. After we obtained the starting conformations, we performed MD runs for 1 μs with a 2 fs time step under a constant pressure of 1 atm. Conformations were saved every 20 ps. In the MD runs, weak constraints of 1*kcal*/*mol* on hydrogen bonds for the ending bases were added to the system in order to reduce artificial fraying at the ends.

We also performed a test run with the same method and procedure as mentioned above for an experimentally obtained structure of DNA triple helix with protonated cytosine whose PDB ID is 1BWG (68). After a 1 μs MD simulation, we found that the triplex was stable and very close to the experimental conformation. To further investigate the stability of selected structures, we also performed MD runs under higher temperatures with all other computational settings being the same as above.

## RESULTS

### Initial modeling of the DNA triple helices

**Figure 1.**
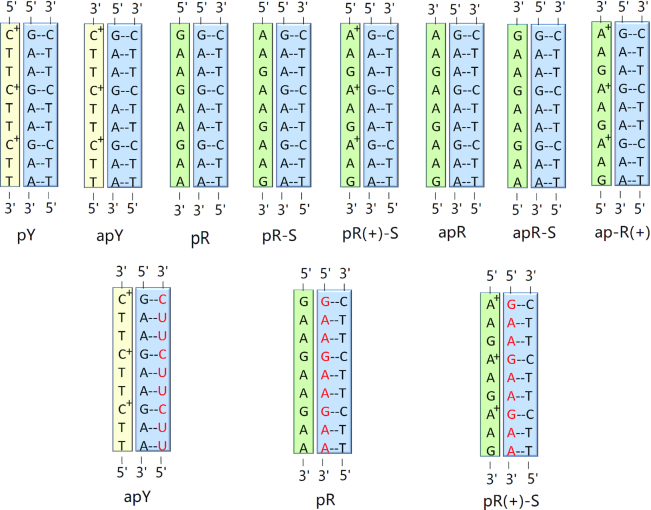
Scheme of the non-equivalent triplexes simulated in this work. Watson–Crick duplexes are highlighted in blue boxes, pyrimidine third strands in yellow boxes, and purine third strands in green boxes. Top row: eight pure DNA triplexes (for each cartoon, two triplexes were studied, with the third strand either in all anti or in all syn conformations). Bottom row: hybrid RNA/DNA triplex with the RNA strand (red) forming part of a hybrid Watson–Crick duplex. The labeling notation is shown below each triplex.

The triplexes consist of a regular B-DNA GAA/TTC helix with standard Watson–Crick basepairing, and a third all purine (R) GAA or all pyrimidine (Y) TTC strand, that is placed in the major groove of the B-DNA helix, as steric clashes would prevent its placement in the minor groove. This allows for 8 possible triple helices as shown in Figure 1, where the terms parallel or antiparallel refer to the orientation of the third strand with respect to the GAA strand of the GAA:TTC B-DNA duplex. The assumption that the third strand bonds with the purines of the helical duplex is based on the traditional Hoogsteen base triads (69), where the middle base in the triad is always a purine, as this maximizes the number of hydrogen bonds. For the TTC·GAA:TTC triplex, we considered both protonated and unprotonated cytosines in the third strand. We found that the unprotonated antiparallel case (apY in the notation described below) was completely unstable (Supplementary Figure S6); and the parallel case (pY) was marginally stable (Supplementary Figure S7), certainly less stable than its protonated case. Clearly, the protonation of the cytosine allows for the formation of hydrogen bonds with the guanines of the GAA strand in the B-DNA duplex. From now on, we will only discuss the protonated cases. The protonation of the cytosines is supported by experimental evidence (35,69–72), both for a parallel or antiparallel cytosine third basis. This results in two cases that can form hydrogen bonds: the protonated pyrimidine third strand in either parallel or antiparallel direction (the latter shifted by one base). For the all-purine third strand GAA·GAA:TTC triplex there are 6 cases. The GAA third strand can be parallel or antiparallel, and can be shifted as shown in Figure 1. In addition, the adenine can be protonated, as it has been noticed that the protonated adenine can form good hydrogen bonding structure with the G:C base pair (73). Thus, we proposed two semi-protonated mismatched R·R:Y triple sequences (purine-parallel-protonated-shifted and purine-antiparallel-protonated) to investigate the potential stability and structure of the mismatched triplex sequences. This gives a total of 8 different structures, as shown in Figure 1. For each of the triplexes in Figure 1, we considered two conformations for the third strand, which differ in the value of the glycosidic angle, such that the nucleotides in the third strand are all in an *anti* or all in a *syn* conformation. The rationale behind this is that *syn* conformations are found in mismatches of trinucleotide repeats (50,52,54,74–78). Thus, we have a total of 16 different initial conformations. In order to refer to them, we introduce the following notation. When the third strand is TTC or GAA we use the notation Y or R. The C’s in the TTC third strand are always protonated, as discussed above (so no need of extra notation). On the other hand, one of the A’s in the GAA third strand can be neutral or protonated in order to form A^+^ ·G–C triple base planes with the G–C Watson–Crick pairs of the B-DNA duplex. In this case, we use the R(+) notation when A’s in the GAA third strand are protonated. For parallel and antiparallel third strands, we precede the Y, R notation by ‘p’ or ‘ap’, while if the strand is shifted, the notation ends with ‘-S’. Finally, the anti or syn conformations of the glycosidic angle are denoted by (a) or (s). Thus, a triple helix where the third strand GAA is parallel and shifted, and every first A in GAA is protonated, with all the bases in anti conformation, is abbreviated as pR(+)-S(a).

**Figure 2.**
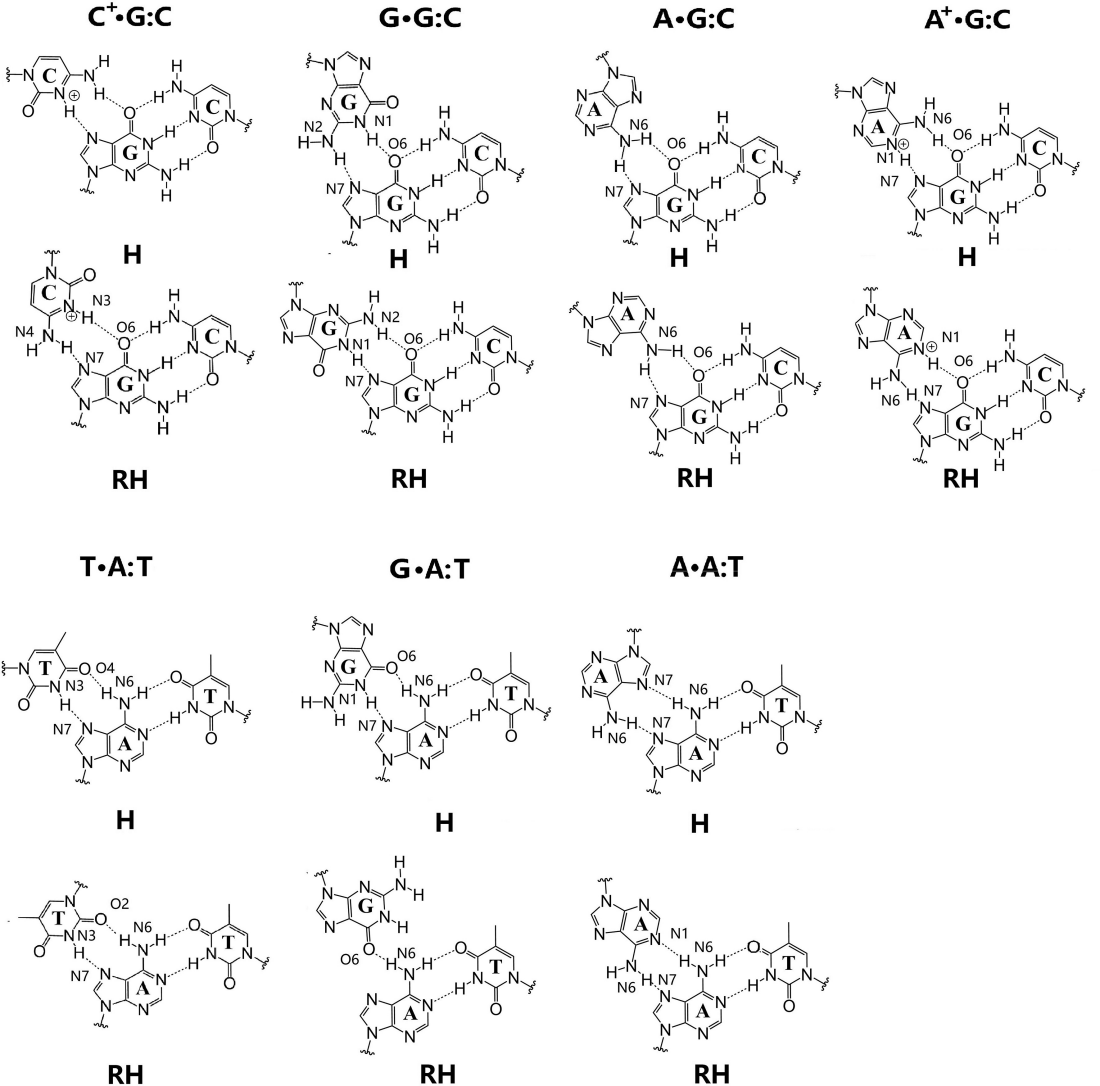
Initial hydrogen bond patterns for all the triple helix sequences.

The 16 triplexes involve hydrogen bond patterns that have traditionally been identified as Hoogsteen or reverse Hoogsteen hydrogen bonds, and patterns that have not been reported previously which we identify as similar to those reported. Thus, we define ‘type H’ hydrogen bonds as those bonds that represent traditional Hoogsteen bonds or Hoogsteen-like hydrogen bonds, and ‘type RH’ hydrogen bonds as those bonds that represent traditional reverse Hoogsteen bonds or reverse-Hoogsteen-like bonds. These are displayed in Figure 2 and Table 1.

**Table 1.** Summary of nucleotide triplets and their corresponding conformational counterparts

Triple step type	Corresponding triple sequence
H-type *C*^+^·*G*:*C*	pY(a) apY(s)
RH-type *C*^+^·*G*:*C*	pY(s) apY(a)
H-type *G*·*G*:*C*	pR(a) apR-S(s)
RH-type *G*·*G*:*C*	pR(s) apR-S(a)
H-type *A*·*G*:*C*	pR-S(a) apR(s)
RH-type *A*·*G*:*C*	pR-S(s) apR(a)
H-type *A*^+^·*G*:*C*	pR(+)-S(a) apR(+)(s)
RH-type *A*^+^·*G*:*C*	pR(+)-S(s) apR(+)(a)
H-type *T*·*A*:*T*	pY(a) apY(s)
RH-type *T*·*A*:*T*	pY(s) apY(a)
H-type *G*·*A*:*T*	pR-S(a) pR(+)-S(a)
	apR(s) apR(+)(s)
RH-type *G*·*A*:*T*	pR-S(s) pR(+)-S(s)
	apR(a) apR(+)(a)
H-type *A*·*A*:*T*	pR(a) pR-S(a)
	pR(+)-S(a) apR(s)
	apR-S(s) apR(+)(s)
RH-type *A*·*A*:*T*	pR(s) pR-S(s)
	apR(+)-S(s) apR(a)
	apR-S(a) apR(+)(a)

To better understand the relation between these hydrogen bond patterns, we can define pairs of triple helices that are related by certain symmetries. We define two triplexes as being *conformational* counterparts when they share the same triple base steps but the third strand has the opposite direction and the glycosidic angles of its bases are flipped, such as pY(a) and apY(s) triplexes (notice that the pyrimidine antiparallel strands are shifted in order to hydrogen bond with the B-DNA duplex). Such conformational counterparts share the same initial hydrogen bond pattern. This can be understood by considering Figure 2. As an example, consider the middle T·A:T base triplet in the case of pY(a). Flipping the direction of the third strand without changing the glycosidic angle, destroys the T–O4 hydrogen bond because the O4 atom in the third-strand T shifts away from the N6 atom of the A basis that belongs to the B-DNA duplex. However, by rotating the basis by 180° toward the syn conformation brings back the O4 atom and restores the hydrogen bond. Thus, the simultaneous operations of inverting the third strand direction and flipping the glycosidic angles of its bases by 180° leaves both the H-type and HR-type bonds unchanged. In addition, we define two triplexes as being *directional* counterparts when one of them is type H and the other is type RH. Thus, changing only the direction of the third strand in the pY(a) destroys the T-O4 and A-N6 hydrogen bond, but a new T–O2 and A–N6 hydrogen bond (type RH) forms; pY(a) and apY(a) are directional counterparts.

The initial triple helices were built starting with a standard B-DNA GAA/TTC duplex. For the third strand, we built another B-DNA duplex containing the sequence of the third strand, which in turn was isolated as a single strand as shown in Supplementary Figure S1. This third strand was then moved into the major groove of the GAA/TTC helix using the molecular editor Avogadro (79). Finally, for the *syn* conformations, we rotated the bases on the third strand by 180°. These primitive models required adjustments that were achieved via MD with constraints through a 1 kcal/mol harmonic potential enforcing the hydrogen bond patterns described above (Figure 2 and Table 1) during equilibration. These constraints were eliminated at the start of the regular constant pressure MD simulations. A few hundred nanoseconds into the regular MD, some structures maintained the hydrogen bond patterns, while others evolved to form different hydrogen bond patterns that proved to be stable, and others just became unstable.

### Initial modeling of the hybrid triple helices

For the DNA·RNA:DNA hybrid triplexes, we only consider the three most probable cases for the simplest geometry of the R-loop (without folding back of the single strands). First, notice that the third strand will still form hydrogen bonds with the purine strand (DNA or RNA) of the hybrid duplex, for the same reasons as explained before. Second, we will assume that the two strands of DNA in the R-loop continue being antiparallel (i.e. discard possible folding back events), and therefore the two DNA strands in the DNA·RNA:DNA triplex are antiparallel. That leaves only two cases: if the template DNA strand is GAA or TTC, then the third DNA strand is TTC *antiparallel* (to the DNA GAA purine strand), or GAA *parallel* (to the RNA GAA purine strand). They can be denoted as d(TTC^+^) ·d(GAA):r(UUC) (apY) and d(GAA)·r(GAA):d(TTC) (pR). We consider the C’s in the third TTC DNA strand to be protonated. In addition, we consider a third case where one of the A’s of the GAA third strand is protonated in order to form A^+^–G pairs with the RNA G’s in the hybrid duplex. This gives rise to the pR(+)-S case, according to our previous notation. Finally, we only consider anti conformations for the bases in the hybrid triplex.

The initial hydrogen bond patterns for the hybrid triple helices are exactly the same as the corresponding ones for pure DNA triple helices. For each triplex we consider two initial conformations: one where the hybrid RNA:DNA duplex starts from an ideal B-DNA conformation, and the third DNA strand is also added in B-DNA conformation; and one where both the hybrid duplex and third strand are in A-DNA conformation.

### New metrics for the stability analysis of the triple helices

During the simulations, we observed that the GAA/TTC DNA duplex part of the triplex is very stable at 300 K in all cases, and therefore measuring the triplex stability simply comes down to quantifying the stability of the third strand with respect to the helical duplex. We define new quantities to better characterize the presence of the third strand. From the structural point of view, the two most important quantities to stabilize the third strand are the hydrogen bonds that it forms with the B-DNA duplex and the π–π stacking interaction between the bases of the third strand. Simply counting the total number of hydrogen bonds that the third strand forms with the duplex does not result in an accurate characterization of the triplex stability. As Supplementary Figure S2.a shows, one can conceive of cases with a high number of hydrogen bonds that link bases on different planes, which do not, however, result in an increased triplex stability. We therefore define the hydrogen bond number for a third strand in a triple helix as:

(1) $$\begin{equation*} H^{{\rm eff}}=\Sigma H^{{\rm eff}}_i \end{equation*}$$

where $H^{{\rm eff}}_i$ is the number of hydrogen bonds that a base on the third strand forms with the bases of the duplex on the same plane, as shown in Supplementary Figure S2.c. This avoids counting inter-plane hydrogen bonds that destabilize the triplex geometry.

Compared to the simple definition of hydrogen bonds, quantifying the π–π stacking interaction is far more complex (80). One popular way to quantify it is to calculate the value of overlap area between the adjacent bases as applied by 3DNA (81). However, for the third strand in the triple helix this method may not work so well, as shown in the extreme example of Supplementary Figure S2.b, where all bases are perfectly stacked but not linked to the duplex. We notice that according to first principles calculations, the stacking of DNA bases is not the sandwich stacking presumed by the maximum overlap area, but a parallel-displaced stacking whose stacking interaction is influenced by relative shift and twist (82,83). Also, it is noted that there exists an inter-strand stacking interaction, which is as important as intra-strand although it needs no overlap area (84). As the stacking mechanism of DNA bases is quite complicated, we propose a mean-field approximation method to roughly quantify the π–π stacking interactions of the third strand in a triplex. First, we calculate the effective area of each step in the third strand:

(2) $$\begin{equation*} A^{{\rm eff}}_i=A^0_i(\mathrm{cos}\;\alpha _i-\mathrm{sin}\;\alpha _i) \end{equation*}$$

where $A^0_i$ is the area of the aromatic ring(s) of the *i*th third-strand base in the triplex (based on their molecular structure, *A*^0^ is estimated to be 4.95 Å^2^ for T and C, and 8.29 Å^2^ for A and G) and α_*i*_ is the angle between the normal vector of the third-strand base and the normal vector of the Watson–Crick base pair at the *i*th step. The cos α represents the effect of the parallel stacking as it projects the aromatic ring area onto the horizontal plane of the B-DNA Watson–Crick base pairs while the subtracting sin α term penalizes the T-shape stacking (strongly disliked by DNA), as it projects the ring area onto the vertical plane. We set the cutoff value for α_*i*_ to 45°, if α_*i*_ > 45°, then $A^{{\rm eff}}_i$ is set to be zero. In addition, because of the essential role of hydrogen bonds in the π-π stacking of base pairs (85), we force $A^{{\rm eff}}_i$ to be zero if $H^{{\rm eff}}_i$ is detected to be zero. This procedure ensures that the base flipping observed in Supplementary Figure S2.b does not contribute to the effective stacking interaction of our algorithm. After we get the values of the effective area of each step, the effective stacking interaction between the *i*th and the (*i* + 1)th step is calculated as:

(3) $$\begin{equation*} S^{{\rm eff}}_i=\sqrt{A^{{\rm eff}}_i\cdot A^{{\rm eff}}_{i+1}} \end{equation*}$$

The effective total stacking area is given by:

(4) $$\begin{equation*} S^{{\rm eff}}=\Sigma S^{{\rm eff}}_i \end{equation*}$$

A schematic procedure of our algorithm is shown in the right panel of Supplementary Figure S2.c.

### Molecular dynamics of the DNA triple helices

**Figure 3.**
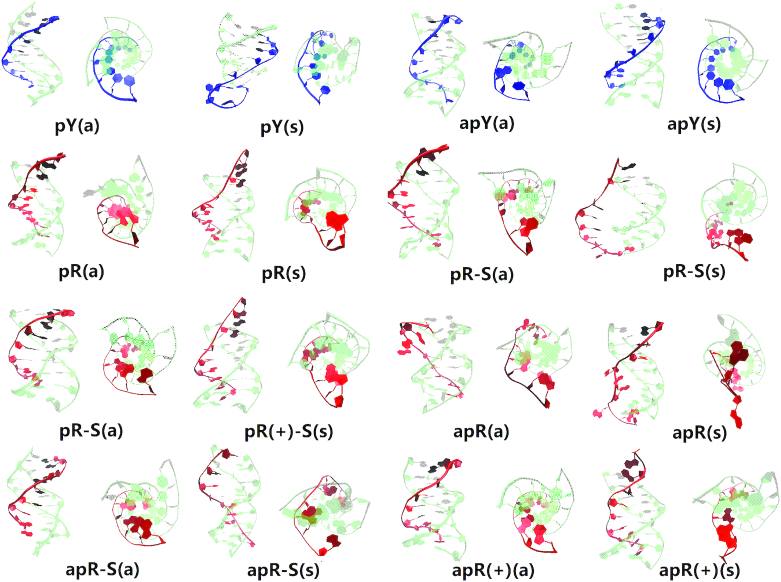
Snapshot of the conformations of the 16 pure DNA triplexes at 1 μs. The third strand of the triplex is colored either blue (pyrimidine) or red (purine) while the duplex part is colored green.

Figure 3 shows a snapshot of the final structures obtained after 1 μs MD runs for all the 16 triplex cases proposed. We colored the Watson–Crick duplex part as light green while the third strand is colored either blue (pyrimidine) or red (purine). The side view allows us to see the attachment (or lack thereof) of the third strand to the B-DNA duplex through intra-step hydrogen bonds, while the top view gives an idea of the base stacking. The conformations after 1μ*s* clearly show the structures that are unstable, as indicated by the detachment of the third strand. The first row in the figure shows that both the pY(a) triplex and its conformational counterpart, the apY(s) triplex are stable, with a well preserved triple helix structure after 1μ*s* MD simulations. It is well known that in the syn conformation the nucleotide bears a strong torsion because of the steric repulsion associated with the sugar ring. However, the structure of the apY(s) triplex is stable after 1 μs due to the stabilization from hydrogen bonds and stacking interactions that overcome the destabilization caused by the χ torsion at a temperature of 300 K. This is not the case for the pY(s) triplex, whose final structure is severely deformed. Its conformational counterpart, the apY(a) triplex, looks stable with some small deformation.

**Figure 4.**
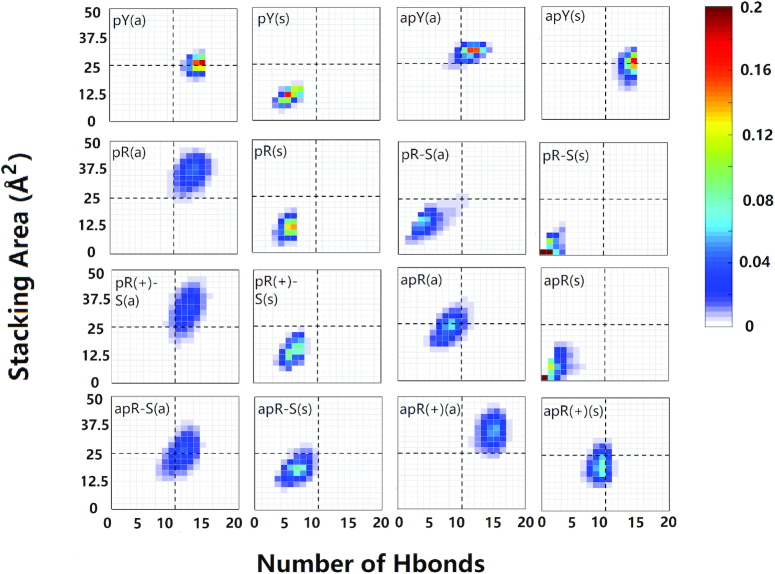
Two-dimensional histograms of the effective stacking area versus the effective hydrogen bond number as obtained from the last 800 ns of the MD simulations for the 16 DNA triplexes.

There are 12 cases with purine as the third strand (the R·R:Y triplexes). Figure 3 shows clearly unstable cases; the four stable cases seem to be pR(a), pR(+)-S(a), apR-S(a), and apR(+)(a). To get a better statistical description that goes beyond the final structures, we plot in Figure 4 the effective stacking area versus the number of hydrogen bonds as defined in the previous section. The distributions have been computed over the last 800 ns. More stable triplexes have higher values in both functions, and therefore better distributions tend to be located in the upper right quadrant. This statistical analysis agrees with the qualitative description provided by the final triplexes. For Y·R:Y, all but the pY(s) triplex seem to have comparable stability. Results for R·R:Y confirm what the final structures suggested: the more stable cases are pR(a), pR(+)-S(a), apR-S(a) and apR(+)(a).

**Figure 5.**
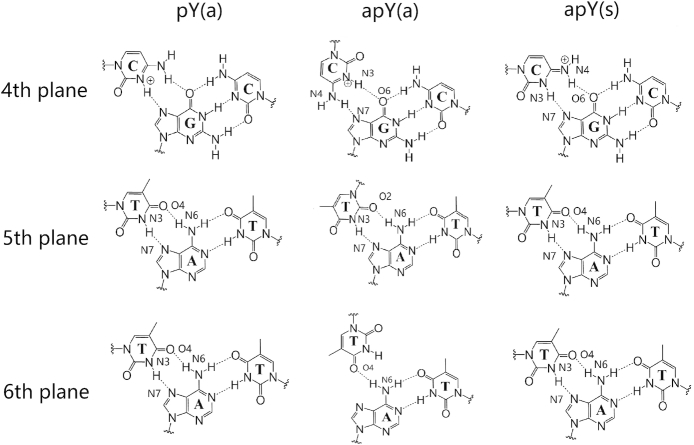
Dominant hydrogen bond patterns for the middle base planes of stable Y·R:Y triplexes.

**Figure 6.**
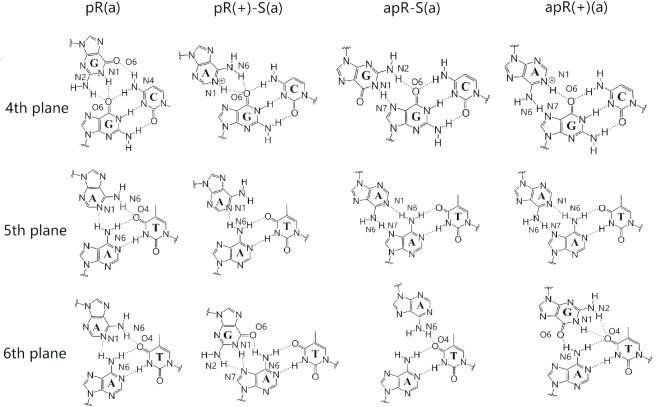
Dominant hydrogen bond patterns for the middle base planes of stable R·R:Y triplexes.

As the triplexes evolve, initial hydrogen bond patterns tend to change as shown for the inner steps in the triplexes in Supplementary Figures S3–S5, where dynamically coexisting hydrogen bonds are displayed. Figures 5 and 6 show the most commonly observed hydrogen-bond patterns that are different from the initial patterns for the inner base triplets in the most stable triplexes. The Y·R:Y (Figure 5) cases are simpler. For the conformational type H counterparts, pY(a) and apY(s) triplexes, the initial hydrogen bond patterns are preserved. The type RH apY(a) triplex shows fluctuations on the fourth plane (Supplementary Figure S3) with the initial pattern being more populated; the 5th plane remains the same, and the 6th plane loses one hydrogen bond.

For the stable R·R:Y triplexes we observe more variations in the hydrogen bond patterns (Figure 6). To describe the change in hydrogen bonds, we define a notation such that the first atom in the bond belongs to the third strand while the second atom belongs to the B-DNA duplex. First consider the pR(a) triplex: on the fourth plane G·G:C the stable hydrogen bond pattern is different from our initial guess. The initial N1(G)–O6(G) and N2(G)–N7(G) bonds are replaced by O6(G)–N4(C) and N1(G)–O6(G) which coexists with a more stable bifurcated version [N1(G)–O6(G) and N2(G)–O6(G)]. On the fifth and sixth planes, the most stable hydrogen bond pattern corresponds to the adenine in the third chain re-orienting itself so as to form hydrogen bonds with both the A and T bases in the B-DNA duplex [N6(A)–O4(T) and N1(A)–N6(A)]; this pattern coexists with slightly fewer stable patterns where one of those bonds is broken (Supplementary Figure S4).

Now consider the pR(+)-S(a) case (type H). The stable structure of the 4th plane is different from our initial guess, as the N1(A+)–N7(G) is replaced by N1(A+)–O6(G). However, our initial guess is still present as one of the coexisting patterns, while the other coexisting pattern loses the N6(A+)–O6(G) initial bond (Supplementary Figure S4). For the fifth plane, the more stable structure differs from our initial guess as the N6(A)–N7(A) and N7(A)–N6(A) bonds are replaced by an N1(A)–N6(A) bond; for the other important coexisting structure the hydrogen bonds link the two bases in the B-DNA duplex: N1(A)–N6(A) and N6(A)–O4(T) bonds. For the 6th plane, our initial guess still holds as a coexisting structure; in the most stable structure the original N1(G)–N7(A) bond becomes a more stable, bifurcated bond with the addition of an N2(G)–N7(A) bond.

The next case to discuss is apR-S(a) (type RH, Figure 6 and Supplementary Figure S5. Our guess structure is the final stable structure for its fourth and fifth planes. The most common alternative structure of fourth plane is given by the substitution of N2(G)–O6(G) by N2(G)–N7(G) while that of the fifth plane is simply the vanishing of all hydrogen bonds. For the sixth plane, our initial guess is one of the most common coexisting structures. The most stable hydrogen bonding structure displays only one hydrogen bond, N6(A)–O4(T), which may be due to a base stacking effect. Similarly to the fifth plane, the loss of all hydrogen bonds also results in a coexisting pattern on the 6th plane.

Finally, we consider the apR(+)(a) case (type RH, Figure 6 and Supplementary Figure S5). For the 4th plane, our initial guess is also the most stable structure, which coexists with two other patterns: one loses the N1(A+)–O6(G) hydrogen bond, while the other forms the N1(A+)–N7(G) bond in place of the N1(A+)–O6(G) bond. The fifth plane preserves our initial guess. On the sixth plane, the most stable pattern augments the initial O6(G)–N6(A) bond with two additional hydrogen bonds, N7(G)–O4(T) and N2(G)–O4(T), which make the pattern more energetically favored. In the most important coexisting structure, one of these bonds is absent.

In order to determine the conformations that are probabilistically more stable, we consider again the possible candidates. Out of the three Y·R:Y cases that show stability, we believe that the case where the pyrimidines in the third strand are in syn conformation represents a long-lived metastable state where the unfavorable χ torsion is stabilized by an initial configuration that maximizes hydrogen bonds and stacking interactions (in other words, if such a state could be set up experimentally, the resulting conformation would also prove to be long-lived). Discarding such configuration leaves two candidates that show stability in the 1μ*s* time scale: the pY(a) and the apY(a). None of the R·R:Y cases shows stability for the syn conformations in the third strand. As a further test of stability, we carried out higher-temperature MD simulations. Final configurations at 300*K* were chosen as a start for these 1μs simulations which entail 0-400 ns at 320 K, 401–1000 ns at 340 K and 1001–1400 ns at 360 K. We obtained an average structure based on the late conformations at 300 K and calculated the RMSD of the higher-temperature runs with respect to the average 300 K conformations, as shown in Supplementary Figure S8. The difference between the two pyrimidine directional counterparts is appreciable: the conformations for the apY(a) triplex seem to undergo more fluctuations and perhaps some conformational instability compared to pY(a) as the temperature increases. With respect to the purine case, the RMSD results for the higher-temperature simulations indicate possible lower stability for apR-S(a), and comparable stability for pR(a), and the two protonated cases: pR(+)–S(a) and apR(+)(a).

Finally, these triplex simulations were run under the presence of Mg^2 +^ ions in the solution. In experiments, Mg^2 +^ ions play two roles: first, they lower the free energy barriers for the assembly of secondary structures; and second, they stabilize the newly formed secondary structures. Since our simulations start with triplexes already formed as initial conditions, the first role of the ion is irrelevant, but the presence of the Mg^2 +^ ions could induce changes in stability. In these new simulations we used a concentration of 200 mM, which is approximately 10 times or more the physiological concentration. We found that the presence of Mg^2 +^ ions preserves the relative stability of the triplexes as described above with only Na^+^ ions (with minor variations in the statistical graphs) except for the apR(a) triplex, that was unstable in absence of the Mg^2 +^ ions but becomes stable in their presence (Figure S9 in Supplementary). So, this particular triplex was simulated again under 40 mM MgCl_2_ and 80 mM MgCl_2_ concentrations, which are still considerably larger than physiological concentrations. We found that under these reduced Mg^2 +^ concentrations, the triplex is only marginally stable (after careful equilibration, some runs remain stable but the structure unravels in other runs). Therefore, once the triplexes are formed, the presence of Mg^2 +^ ions does not affect the stability results obtained with only Na^+^ ions, unless extremely high divalent concentration are used.

Next, we briefly describe some other structural features of the triplexes. A triple helix has three grooves whose widths are mainly determined by the nature and orientation of the third chain and the hydrogen bond patterns. Charged or neutral bases do not alter the widths of the grooves. For the parallel chains, the narrowest groove takes place between the parallel third strand and the GAA strand of the duplex. This is followed in order of increasing width by the original minor groove in the duplex, and last by the groove between the third chain and TTC chain in the duplex (the latter is wider for the Y-third strand than for R-third strand). For the antiparallel third strand, the narrowest groove is between the antiparallel third chain and the TTC strand of the duplex. The groove between the third strand and the GAA strand of the duplex and the original minor groove of the duplex are wider and approximately the same. With respect to ion distributions, the Na^+^ ion density in these simulations is relatively small. Supplementary Figure S10 shows the ion distribution for the pY and pR triplexes for the last 200 ns. As it can be seen in the figures, most ions concentrate in the very electronegative, narrow groove between the third strand and the GAA strand of the duplex. There are considerably fewer ions in the groove between the third strand and the duplex TTC strand, and extremely few in the original minor groove of the B-DNA duplex. Typical parameters for DNA duplexes that form part of the triplexes pY and pR(+)-S are shown in Supplementary Figure S11. Average values for the last 200 ns of the simulations for twist, roll, helical rise, inclination, slide, and *Z*_*p*_ for the Watson–Crick duplex part of the pR, pY, pR(+)-S and apR(+) triplexes are: twist, 31.3°, 30.9°, 31.1°, 33.2°; roll, 1.24°, 1.49°, 2.52° and 2.89°; helical rise, 3.35, 3.28, 3.24, 3.28 Å; inclination, 2.41°, 2.62°, 4.44°, 4.98°; slide, –1.21, –1.18, –1.33, –0.90 Å; and Zp, 0.32, 0.13, 0.43, 0.00 Å. We see in these values a mixture of B- and A-DNA features. Twist values show that attaching a third strand to the GAA/TTC duplex slightly unwinds it, especially with the third strand in parallel position, making the duplex more A-DNA as far as twist goes. Average inclination values all assume small positive values, closer to B-form. Helical rise and small values of roll put the duplex closer to B-form, while the negative slide is more characteristic of the A-DNA. Finally, *Z*_*p*_ values less than 0.5 put these duplexes into the B-DNA camp.

### Molecular dynamics of the hybrid helical duplexes

**Figure 7.**
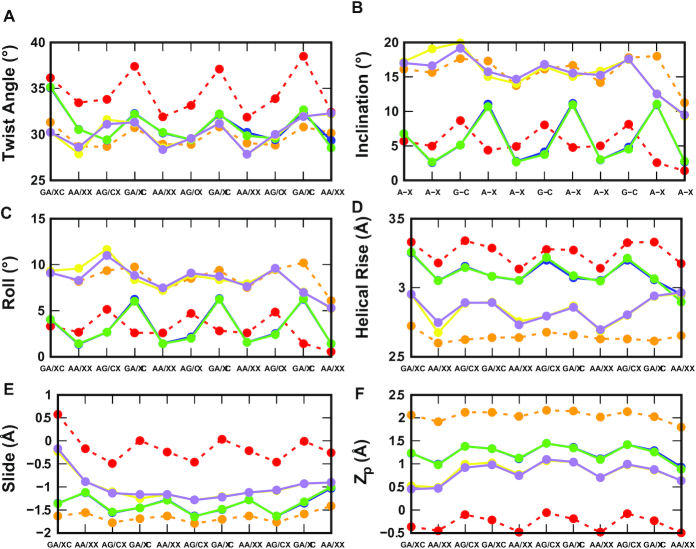
Average basepair inclination, and basepair-step twist, roll, helical rise, slide and *Z*_p_ of double helices. Red: d(GAA):d(TTC) (B-DNA); orange: r(GAA):r(UUC) (A-RNA); blue and green: r(GAA):d(TTC) starting from ideal B-DNA (blue) or ideal A-RNA (green); yellow and purple: d(GAA):r(UUC) starting from ideal B-DNA (yellow) or ideal A-RNA (purple). Data was averaged over the last 200 ns. ‘X’ in the x-axis labels stands for either T or U, according to the sequence.

There are two types of hybrid duplexes that are not equivalent: RU=d(GAA):r(UUC) and DT=r(GAA):d(TTC). For each of these hybrids, we started the simulations with ideal B-DNA and A-RNA conformations. Convergence of the simulations is confirmed by the convergence of these initial duplexes to a final duplex that is independent of the initial conditions. This convergence can be appreciated in the analysis of structural parameters presented in Figure 7, which also make evident the periodicity of the three different steps of the sequence. Supplementary Figure S12 shows the RMSD of the four hybrid duplexes. Clearly these duplexes are somewhere between A-RNA and B-DNA, but closer to A-RNA. In particular, the average RMSD over the last 200 ns of the simulations are: for RU, about 1.97Å with respect to A-RNA and 2.57 Å with respect to B-DNA; and for DT, about 1.56 Å with respect to A-RNA and 1.92 Å with respect to B-DNA. Thus, DT is closer to both A-RNA and B-DNA than RU is to either form. Two main factors contributes to relative stability: hydrogen bonds and stacking area. With respect to the hydrogen bonds, the most important difference is given by the d(A):r(U) basepair that is extremely weak (86). With respect to the stacking area of the base steps, we used 3DNA (87) in order to compute the total stacking area of inner steps 2 to 8 and found this to be ≃48 Å^2^ for DT and ≃43 Å^2^ for RU. Thus, DT displays better stability than its RU counterpart. Interestingly DT=r(GAA):d(TTC) seems to achieve this stability by combining stabilizing features from A- and B-forms: rise, inclination, and roll, among other parameters, are closer to B-DNA than A-RNA, while the weaker RU=d(GAA):r(UUC) duplex is closer to A-RNA as measured by these parameters. Instead, the more discriminatory parameters (88) slide and the *Z*_p_ parameter, defined as the mean z-coordinates of the backbone phosphorus atoms with respect to individual dimer reference frames (89), are closer to A-RNA, as shown in Figure 7.

### Molecular dynamics of the hybrid triple helices

**Figure 8.**
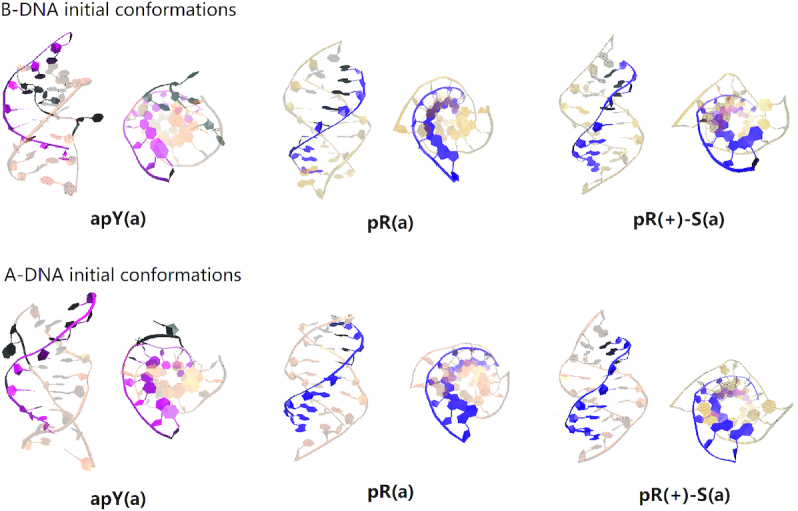
Snapshot of the conformations of the 6 hybrid DNA/RNA triplexes at 1 μs. The RNA strand of the triplex is colored either pink (pyrimidine) or violet (purine) while the DNA strands are colored light orange. Bases that have lost all hydrogen bonds or are flipping out are indicated in black shadow.

**Figure 9.**
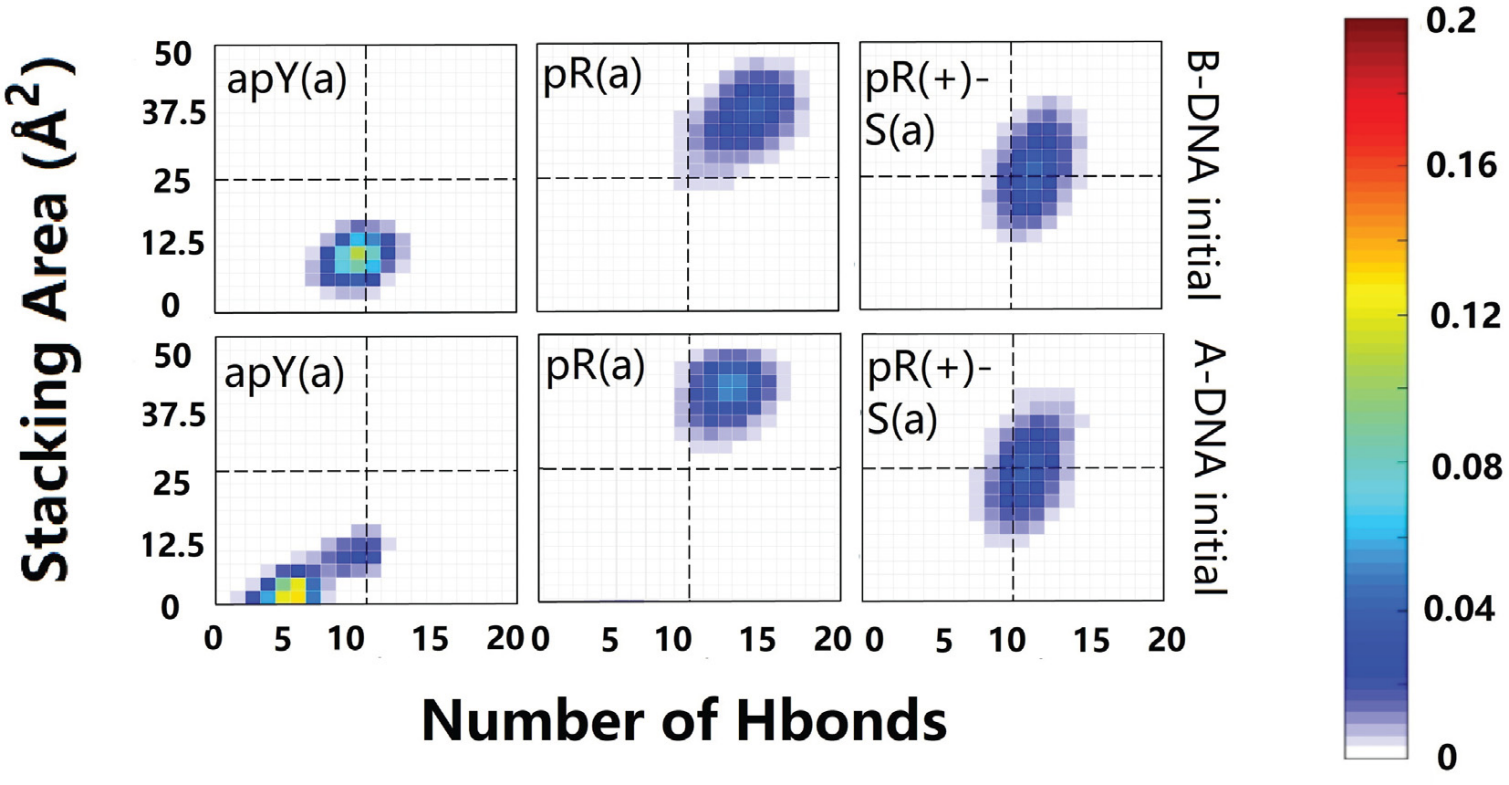
Two-dimensional histograms of the effective stacking area versus the effective hydrogen bond number as obtained from the last 800 ns of the MD simulations for the six hybrid DNA/RNA triplexes.

As discussed in the Initial Modeling section, the simplest collapsed R-loop (without back-folding of the single strands) can only form three hybrid triplexes. Our simulations start with either an ideal B-DNA conformation or an ideal A-DNA one. In real life, one expects the duplex part of the triplex, like the hybrid helical duplexes described above, to be somewhere in the spectrum between these two ideal structures. In the ideal A-DNA triplex, the third strand ends further away from the duplex than in the ideal B-DNA conformation, and therefore the initial fully A-DNA conformation in the pR triplex (but not in the pR(+)-S triplex) is unstable because the third strand detaches before the duplex has time to equilibrate. Therefore, we ran an initial equilibration step where the third strand is constrained to remain attached to the duplex while it equilibrates, and this became the new A-like initial conformation. Final conformations for the six cases are shown in Figure 8 (where bases that have flipped out are colored in black). The statistical analysis based on the effective hydrogen bonds and the effective stacking areas is shown in Figure 9. The results shown in these figures indicate that when the DNA third strand is formed by the pyrimidines, the resulting apY triplexes are unstable: bases are flipping out not only in the third strand but also in the RNA strand that forms the hybrid duplex. On the other hand, the pR and pR(+)-S triplexes are stable and their initial A- and B-forms converge to the same structure as the statistical analysis in Figure 9 clearly shows, with a strong distribution in the upper right quadrant for pR.

Next, we examine the primary hydrogen bond patterns for the three stable cases. These are shown in Supplementary Figures S13 and S14. For a given sequence, hydrogen bond patterns for the hybrid triplex are the same as those for the pure DNA triplex except for the fifth plane of the pR(+)-S triplex where a new bond between the N6 atom of DNA adenine and the O4 atom of DNA thymine is the dominant isomer in the hybrid triplex and a less populated isomer in the pure DNA triplex. The statistical analysis shows that the pR(+)-S conformations are less stable for the hybrid triplexes than for the pure DNA triplexes: in the 2D histograms, the distributions in the RNA-containing triplexes are displaced downwards and towards the left compared to their counterpart in the pure DNA triplexes. While the pR triplexes settle quickly into their final hydrogen bond patterns, the pR(+)-S conformations show some coexisting isomers after 200 ns, as shown in Supplementary Figure S14.

The Na^+^ ion distribution for the hybrid pR triplex for the last 200 ns is shown in Supplementary Figure S10. In both the pure DNA and hybrid pR triplexes, most of the ion distribution sits in the very narrow groove between the third chain and the GAA strand of the duplex, with negligible distribution in the original minor groove of the duplex. The difference between the two triplexes is that the pure DNA triplex still carries ions in the groove between the third strand and the TTC strand of the duplexes, while for the hybrid triplex these ions almost disappear and most of the distribution is concentrated in the narrow groove. Parameters for the hybrid duplexes r(GAA):d(TTC) as they describe a free-standing hybrid duplex or a duplex part of a triplex are shown in Supplementary Figure S15. These figures as well as the values of the *Z*_p_ parameter calculated as 0.32Å for pR and 0.43Å for pR(+)-S show that the hybrid duplexes that form part of a triplex are more B-like than A-like in comparison to their free-standing counterparts.

## DISCUSSION

One common characteristic underlying all TREDs is the transient formation of atypical, non-B DNA stable secondary structures in the expandable repeats (17,18,23–25). Although knowledge of these structures *per se* is not nearly enough to understand these diseases, it certainly helps to understand the interaction of the TRs with the relevant proteins as one tries to decipher the molecular mechanisms behind the diseases. The GAA/TTC TRs associated with Friedreich’s ataxia are unable to form the hairpins that characterize other TRs: GAA/TTC repeats form DNA triplexes (30–38), traditional R-loops (41–44) or, possibly, collapsed R-loops, i.e. hybrid DNA·RNA:DNA triplexes, which have not been directly measured. In the simplest triplex DNA models, the strand that appears twice in a triplex does so via a single turn, so that it ends antiparallel to itself. This would imply that Y·R:Y triplexes can only be parallel (pY) while R·R:Y triplexes can only be antiparallel (apR or apR-S). However, the other two types of triplexes, apY and pR (or pR-S), are important not only in the context of TFO therapies but also as a natural possibility, as it is increasingly becoming clear that more complicated entities can form at R-loops (57). Supplementary Figure S16 shows an example of pR formation that could happen either intra- or inter-molecularly, as has been proposed to occur, for instance, in plasmids (90). Since one of the driving forces of non-B-DNA formation in the cell is negative supercoiling, these simulations, like FRET, gel electrophoresis, circular dichroism, UV melting, UV absorption and other experiments that use short oligonucleotides, ultimately cannot reflect the structural constrains related to non-B-DNA formation. For instance, a number of experiments using GAA-containing supercoiled plasmids report formation of Y·R:Y DNA triplexes only (32,91–94), but other plasmid experiments suggest the presence of R·R:Y triplexes, in particular as precursors to ‘sticky DNA’ (30,31,90,95–98). Interestingly, R·R:Y triplexes have been proposed to act during DNA replication: Fork reversal could occur at pausing forks when triplex formation occurs between two GAA strands and one TTC strand (99,100) Here we discuss our main results.

### Hydrogen bond patterns and their symmetry; measuring hydrogen bonds and stacking for the third strand

We extended definitions of Hoogsteen and reverse Hoogsteen hydrogen bond pattern to encompass other similar patterns classified as ‘H’ and ‘RH’ in Figure 2 and Table 1. These definitions facilitate the visualization of symmetry properties of the triple helix: *conformational* counterparts share the same triple base steps but the third strand has the opposite direction and the glycosidic angles of its bases are flipped, such as pY(a) and apY(s) triplexes (notice that the pyrimidine antiparallel strands are shifted in order to hydrogen bond with the B-DNA duplex). Conformational counterparts belong to the same ‘type’: the simultaneous operations of inverting the third strand direction and flipping the glycosidic angles of its bases by 180° leaves both the H-type and RH-type bonds unchanged. In *directional* counterpart pairs, on the other hand, one triplex is type H and the other is type RH, such as pY(a) and apY(a). In order to characterize these triplexes and their stability we noticed that standard ways of characterizing hydrogen bonds and base stacking fail to give a true measure when applied to the third strand. Thus, we provided a new protocol to count the hydrogen bonds and new definitions of base stacking that explicitly consider the triplex geometry provided by the third strand. Analysis of final conformations and statistical two-dimensional histograms of the effective base stacking versus the effective number of hydrogen bonds for the third strand allowed us to pick the triplexes that are more stable. In all unstable triplexes but one, instability shows up in the detachment of the third strand while the helical duplex remains stable. In the d(TTC^+^)·d(GAA):r(UUC) hybrid triplex, the detachment of the third strand also disrupts the duplex. Plots of the effective base stacking versus the effective number of hydrogen bonds for the third strand capture the instability in both cases: the only circumstance where this description would fail corresponds to the highly improbable case where the Watson–Crick base pairs break but the third strand forms a duplex with the purine strand with H/HR hydrogen bonding.

### Sixteen non-equivalent DNA triple helices can be assembled from GAA and TTC strands

We have provided physical arguments to figure out all possible triplexes that can be formed with three DNA strands composed of either GAA or TTC repeats. There are 8 resulting triplexes depicted in Figure 1, a number that doubles to 16 if one allows for the possibility of bases on the third strand to be in syn conformation, a state of the glycosidic bond that has been observed in other TRs (50,52,54,74–78). For the TTC·GAA:TTC triplex, protonated cytosines (69–72) in the third strand allow the formation of hydrogen bonds with the guanines of the GAA strand in the B-DNA duplex. This results in only two cases that can form hydrogen bonds plus their syn counterparts. For the all-purine third strand GAA·GAA:TTC triplex, there are six cases (plus the six syn counterparts): the GAA third strand can be parallel or antiparallel, can be shifted or not, and can have completely unprotonated adenines or up to one protonated adenine (73) per repeat in order to form good hydrogen bonding structure with the G:C base pair of the B-DNA duplex (Figure 1). The final conformations and the statistical two-dimensional histograms of the effective base stacking versus the effective number of hydrogen bonds for the third strand leads to the stability ranking of the triplexes.

### DNA TTC·GAA:TTC triple helices: the TTC third strand in parallel alignment, pY(a), is most stable

For the TTC·GAA:TTC triplexes both parallel and antiparallel conformations show good stability when the third strand bases are in anti conformation. Interestingly, the apY(s) triplex also shows stability even though the pyrimidine bases of the third strand are in syn conformation (although less common, pyrimidine bases can be found in syn conformations (74)). In this case, the stabilization from hydrogen bonds and stacking interactions overcome the destabilization caused by the χ torsion at a temperature of 300 K. We believe that this is a metastable state facilitated by the good stacking of the initial conformation. Under progressive higher-temperature simulations, the parallel pyrimidine pY(a) triplex is seen as more stable than its directional counterpart, apY(a), in agreement with experiments (33).

With respect to the hydrogen bonds, the original guesses in Figure 2 for the Y·R:Y triplexes are on cue; the most common variation is the formation of hydrogen bonds between bases belonging to the two pyrimidine strands. These cases, however, are less populated, generally because the new pattern results in the same or fewer number of hydrogen bonds than the original pattern.

Our results agree with experiments. Cytosines need to be protonated in order to form stable triplexes, which generally occurs under low *pK_*a*_* (28). However, cytosines buried in the major groove can be protonated even under p*K*_a_ as high as 8 (101,102). Results of a smFRET study (36) show that the Y·R:Y DNA triplex is stable even under neutral pH, with pY being the most stable pyrimidine triplex, a result that confirms previous findings (33–35).

### DNA GAA·GAA:TTC triple helices: the GAA third strand in parallel alignment, pR(a), as well as the two protonated cases, pR(+)-S(a) and apR(+)(a), are most stable

For the GAA·GAA:TTC triplexes, analysis of final conformations and statistical 2D histograms of the effective base stacking versus the effective number of hydrogen bonds for the third strand, Figure 4, reveals four possibly stable structures out of the 12 initial possibilities. The candidates are pR(a), pR(+)-S(a) and apR(+)(a) and, to a lesser degree, apR-S(a) with a central distribution (not in the upper right quadrant). Under progressive higher-temperature simulations, pR(a) shows better stability than apR-S(a), in agreement with experiments (36). Both protonated variants, pR(+)-S(a) and apR(+)(a) show good stability. In addition, a comparison of the statistical graphs suggests the parallel-purine third strand pR triplex is more stable than its pyrimidine counterpart, the pY triplex.

With respect to the hydrogen bonds, the original guesses in Figure 2 for the R·R:Y triplexes, depart more from the original patterns than the Y·R:Y triplexes. In particular, out of the four candidates, the antiparallel triplexes apR-S(a) and apR(+)(a) preserve the original type RH hydrogen bonds shown in Figure 2 with a small variation on the sixth plane. However, the two parallel stable candidates, pR(a) and pR(+)-S(a), which are both type H, seem to undergo considerable re-arrangement of their hydrogen bonds (compare Figures 2 and 6). Additional stability in pR(a) is gained through the formation of hydrogen bonds that join the purine in the third strand not only to the purine but also to the pyrimidine of the B-DNA duplex. With respect to the triplex geometry, the widths of the three grooves are mainly determined by the orientation of the third chain and the hydrogen bond patterns. For the parallel chains, the narrowest groove takes place between the parallel third strand and the GAA strand of the duplex, followed by the original minor groove in the duplex, and last by the widest groove between the third chain and TTC chain in the duplex. For the antiparallel third strand, the narrowest groove is between the antiparallel third chain and the TTC strand of the duplex, and the other two wider grooves are approximately the same. The very narrow groove in the parallel triplexes strongly attracts Na^+^ ions, with most (but not all) of the cation density localized there (Supplementary Figure S10), and almost no ions in the original minor groove of the duplex. Typical parameters for Watson–Crick duplexes that form part of the triplexes display a mixture of B- and A-DNA features. Attaching a third strand to the GAA/TTC duplex slightly unwinds it, especially with the third strand in parallel position, making the duplex more A-DNA as far as twist goes. Average inclination, helical rise and roll depart from ideal B-DNA values, but still are closer to B- than A-DNA. Negative average slides are more characteristic of A-DNA, but *Z*_p_ values <0.5 put these duplexes into the B-DNA camp.

Experimental findings with respect to R·R:Y triplexes are not unanimous. Thermal melting analysis and sedimentation equilibrium analysis (34) as well as smFRET experiments (36) found the parallel GAA-third strand as the most likely conformation. In the first study (34), the complementary repeating regions were linked on the same oligonucleotide (to avoid concentration effects) and the loops flanking the triplex stems may have also played a role in the stability of the triplexes. This study found the parallel Y·R:Y triplex to be more stable than the parallel R·R:Y triplex while the smFRET study suggested that the parallel R·R:Y triplex is more stable than the parallel Y·R:Y triplex. Other experiments involving UV-melting temperature and circular dichroic spectra found that the GAA third strand in antiparallel configuration is more stable (37,38).

The important issue to notice is that although cartoons are presented in experimental studies, showing for instance (36) pY, apY, pR and apR-S conformations (using the notation introduced in our scheme in Figure 1), the fact is that these experiments do not have the resolution to differentiate between pR as reported in their cartoon or pR-S (protonated or not); and apR-S as reported in their cartoons or apR (protonated or not). Both the links between the strands and the insertion of donors/acceptors in the smFRET experiments could allow or force the third strand to slip by just one base to change the alignment. The results of our simulations, which considered all possible combinations, are consistent with these experimental findings. For the Y·R:Y triplex, pY is more stable than apY. For the R·R:Y triplex, we showed that pR is more stable than apR-S and apR. Also, considering that shifting of putative general acid adenine and cytosine p*K*_a_’s toward neutrality has been observed within a molecular context (103,104), we have included protonated adenines in the triplexes, and found that both pR(+)-S and apR(+) triplexes display very good stability. Comparison between the stability of Y·R:Y and R·R:Y triplexes would require to know the state of protonation of the third chain; the base overlap favors the purine third strand and the pR triplex would be more stable than the pY triplex, according to the statistical analysis in Figure 4, which is in line with the smFRET experiments (36). Notice that the apparent contradiction in experimental results about whether the GAA third strand is more stable in parallel or antiparallel conformation may be more a lack of resolution of the structure of the triplex than a contradiction: pR is more stable than apR-S or apR but pR-S is less stable than the two antiparallel versions.

### Hybrid RNA:DNA helices: structural analysis indicates that r(GAA):d(TTC) is more stable and closer to the A-RNA than d(GAA):r(UUC)

We presented results for both possible hybrids, mainly DT=r(GAA):d(TTC) and RU=d(GAA):r(UUC). DT is more stable than RU because of the known fact that the d(A):r(U) basepair is extremely weak (86), and because the average stacking area of the base steps is larger in DT than in RU (∼6.0 Å^2^ versus 5.4 Å^2^ computed with 3DNA). Both hybrids combine A- and B-form features. In particular, in DT rise, inclination, and roll, among other parameters, are closer to B-DNA than A-RNA, while the weaker RU duplex is closer to A-RNA as measured by these parameters (Supplementary Figure S15). However, these parameters *per se* are not enough to determine whether a duplex is closer to A- or B-forms. A survey of high-resolution A and B-DNA oligonucleotides without any modifications showed that of all the parameters used to characterize the structure of double helical DNA, very few have clear discriminating power (88) between A- and B-forms. Slide is one of them, with slide <–0.8 Å in most A-DNA dimer steps and >–0.8 Å in the majority of B-forms. Another parameter (89) is *Z*_p_ that is given by the mean z-coordinates of the backbone phosphorus atoms with respect to individual dimer reference frames: *Z*_p_ is >1.5 Å for A-RNA and <0.5 Å for B-DNA steps. Thus, these discriminatory parameters shown in Figure 7 along with the RMSDs in Supplementary Figure S12 indicate that the duplexes, especially DT, are closer to A-RNA.

These hybrid duplexes can form part of an R-loop or serve as a basis for the DNA·RNA:DNA triplexes. Indeed, the all-purine GAA and all-pyrimidine TTC strands are perfect candidates for R-loops (41,43,44) and, as we have shown, for hybrid triplexes, that can form during bidirectional transcription (45–48). Unlike their triple helical counterparts, hybrid duplexes and their role in R-loops have been more extensively studied in the literature (39,40). Thermodynamic analysis shows that the most stable hybrids form between a purine-rich RNA transcript and the complementary pyrimidine-rich DNA template, and that RNA duplexes are more stable than RNA(R-rich):DNA(Y-rich) hybrid duplexes, which in turn are more stable than DNA duplexes and DNA(R-rich):RNA(Y-rich) hybrid duplexes (42,105–107) (which of the last two is more stable depends on the sequence). Our structural analysis is consistent with this. There is one crystal structure in the PDB for the sequence *r*(GAA-GAA-GAG):d(CTC-TTC-TTC) (108). Parameters reported for this structure are compatible with those presented in Figure 7, with small differences that can be attributed to the different third nucleotide and crystal packing interactions (the authors claim that abutting interactions dominate the packing, with a d(C1):r(G18) pair lying close to the minor groove of a symmetry-related molecule with different abutting interactions to the TTC structure). In this sense, we believe that our simulations provide a better description of both the DT hybrid duplex and the RU hybrid duplex in solution (for the latter, we found no reported structure).

### A rare hybrid DNA·RNA:DNA triple helix (collapsed R-loop): d(TTC^+^)·d(GAA):r(UUC) is unstable while d(GAA)·r(GAA):d(TTC) and d(GA^+^A)·r(GAA):d(TTC) are stable

**Figure 10.**
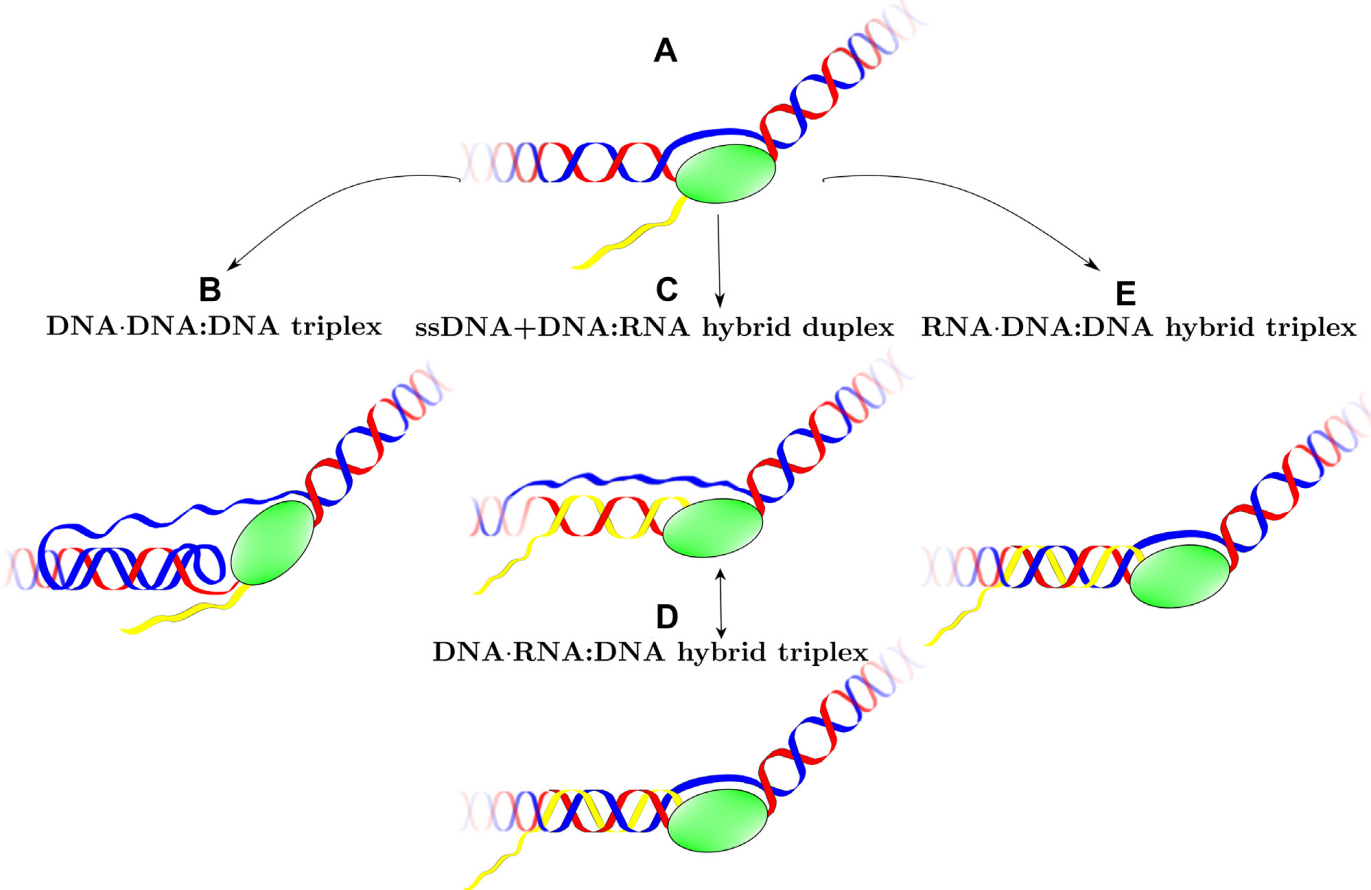
Schematic for the transient formation of non-canonical structures during transcription that results in the stalling of the RNA polymerase (green ellipse). Red strand is template DNA; blue strand is non-template DNA; and yellow strand is mRNA. In the usual physiological transcription, the blue strand would be the coding (GAA) strand, but transcription could also occur bidirectionally in trinucleotide repeats. (**A**) The negative supercoiling wave behind RNAPII opens the transcription bubble giving rise to several possible scenarios. (**B**) The non-template strand could fold back on the DNA duplex and relax the negative supercoiling by winding around the duplex and forming a DNA triplex. (**C**) The mRNA forms a hybrid RNA:DNA duplex with the template DNA strand, while the non-template ssDNA remains unattached in an R-loop. This situation could compete or be in dynamic equilibrium with (**D**): the ssDNA folds back onto the hybrid duplex winding around it to form a hybrid DNA·RNA:DNA triplex or collapsed R-loop. (**E**) Alternatively, the mRNA strand could wind around the DNA duplex to form a hybrid RNA·DNA:DNA triplex. Depending on the extent of the transcription bubble, some of these structures could coexist.

In an R-loop, the hybrid duplex formed by the messenger RNA and the template DNA strand can further hydrogen bond with the third, non-template DNA strand to form a hybrid DNA·RNA:DNA triplex, as shown in the cartoon in Figure 10. If one assumes that during transcription the two strands of DNA in the R-loop continue being antiparallel (i.e. discard possible folding back events), then the two DNA strands in the DNA·RNA:DNA triplex are antiparallel, which limits the number of possible triplexes to three. These are d(TTC^+^)·d(GAA):r(UUC) (apY, that could form during antisense transcription); and d(GAA)·r(GAA):d(TTC) (pR), and the protonated version d(GA^+^A)·r(GAA):d(TTC) (pR(+)-S), both of which can form during sense transcription, shown in Figure 1. In all cases, the third DNA strand mainly bonds with the purine strand (DNA or RNA) of the hybrid duplex. Both the final conformations in Figure 8 and the statistical analysis based on the effective hydrogen bonds and the effective stacking areas in Figure 9 indicate that the apY triplexes are unstable. Compared to its pure DNA apY triplex counterpart that is relatively stable, what makes this hybrid triplex so unstable? Clearly, both triplexes have the same antiparallel DNA third strand and only differ in the hybrid duplex. It has been pointed out before that the stability of triplex DNA is affected by the stability of the underlying duplex (109). We believe that this observation extends to the underlying duplex independent of its DNA/RNA nature: in our case, the d(GAA):d(TTC) DNA duplex is more stable than the d(GAA):r(UUC) hybrid duplex, directly impacting the stability of the resulting triplex with the same third strand. The pR and pR(+)-S triplexes are stable, with their initial A-like and B-like forms converging to the same structures. The better stability of the purine-rich triplexes with respect to the apY ones can be explained in a similar fashion. First, as explained in (E) above, the r(GAA):d(TTC) hybrid duplex is more stable than the d(GAA):r(UUC) duplex. Second, the contribution to stability of the ‘RH’ hydrogen bonds and of the stacking energy of the pyrimidine third chain in the apY case is lower than that for the ‘H’ hydrogen bonds and stacking in the pR cases. In these triplexes, almost all the Na^+^ ion distribution sits in the very narrow groove between the third chain and the GAA strand of the duplex. Parameters for the hybrid duplexes r(GAA):d(TTC) as they describe a free-standing hybrid duplex or a duplex part of a triplex show that the hybrid duplexes that form part of a triplex are more B-like than A-like in comparison to their free-standing counterparts; a pure A-like form would lead to detachment of the third strand.

The formation of a hybrid triple helix, where the ssDNA left behind by RNAPII during transcription is no longer ‘loose’ but attaches through Hoogsteen or reversed Hoogsteen hydrogen bonds to the hybrid DNA:RNA helix, as shown in Figure 10D, was proposed for a different but similar sequence by results of gel electrophoresis experiments (49). The authors of this work called this structure ‘collapsed R-loop’, and gave arguments to support its existence, in spite of direct evidence. In this work, we show that the collapsed R-loop is stable and therefore relevant to the taxonomy of atypical secondary structures related to trinucleotide repeat diseases. Notice that this hybrid triplex is of the form R·R:Y. Earlier studies claimed that RNA strands were excluded from an R·R:Y triple helix for any combination of RNA strands—in other words, out of the eight possible combinations, only the pure DNA triplex could exist (110). These studies were carried out with a very limited set of sequences (only one for the reference cited). In spite of its appeal, this generalization does not hold true. Later bioinformatics and gel electrophoresis studies looking at an RNA third strand binding to a DNA duplex gave a far more nuanced understanding where sequence-dependent effects play a major role (111,112). Indeed, ∼51% of RNA triple forming sequences comprise pure purine (G,A) or mixed motifs (U,G) that bind to R/Y rich duplex DNA (111). This type of hybrid triplex, shown in Figure 10.e, can also form during transcription.

### Biological relevance

Formation of non-canonical structures in the first intron of the frataxin gene interfere with its transcription, drastically reducing the levels of the protein frataxin and causing the pathology associated with Friedreich’s ataxia. A cartoon showing how some of these non-canonical structures can form during transcription is shown in Figure 10, which is a generalization of a scheme previously introduced to interpret results from gel electrophoresis experiments (41). The standing wave of negative supercoiling that follows the RNA polymerase unwinds the two strands of DNA at the transcription bubble (Figure 10A). As proposed before (41), this can free the non-template strand to fall back onto the DNA duplex to form a DNA triplex (Figure 10B). Simultaneously or alternatively, the template strand can be exposed to form a hybrid with the nascent RNA giving rise to the more traditional R-loop (Figure 10C) or to a DNA·RNA:DNA hybrid triplex (Figure 10D), where the two DNA strands remain antiparallel if the transcription bubble is not too large. Alternatively, the nascent RNA could anneal as the third strand in a RNA·DNA:DNA hybrid triplex (Figure 10E). In our work, we showed all the possible structures that a GAA/TTC DNA triplex can have, as well as the probable structures for the rarer collapsed R-loop, DNA·RNA:DNA hybrid triplex. A simple, traditional R-loop formed by a r(GAA):d(TTC) hybrid duplex and the d(TTC) single strand could compete with a parallel d(GAA)·r(GAA):d(TTC) hybrid triplex, shown in our work to be stable, but the less stable d(GAA):r(UUC) hybrid duplex cannot form an antiparallel d(TTC^+^)·d(GAA):r(UUC) triplex, as the latter is not stable. Knowledge of these non-canonical structures and their relative stability can help guide the application of triple-specific antibodies, which have been found to bind triplexes inside the nucleus and to exhibit a higher affinity for RNA–DNA triplexes than for DNA triplexes (38). Recently, a quantitative model of R-loop-forming sequences (RLFS) predicted ≃660 000 RLFS (57), where the meaning of ‘R-loop’ is extended to encompass a whole variety of non-canonical secondary structures that can form when the DNA strands are separated. The authors intended the study to provide a ‘rationale for the discovery and characterization of the non-B DNA regulatory structures involved in the formation of the RNA:DNA interactome’. However, experimental data with molecular resolution for these non-B DNA structures is noticeably scarce. We believe that structural studies such as ours can meaningfully contribute in the creation of such a roadmap.

## Supplementary Material

gkaa665_Supplemental_File
