# Supplementary material for: Atypical structures of GAA/TTC trinucleotide repeats underlying Friedreich’s ataxia: DNA triplexes and RNA/DNA hybrids
Source: Nucleic Acids Res. 2020 Aug 21;48(17):9899–917. doi: 10.1093/nar/gkaa665 (PMC7515735; doi:10.1093/nar/gkaa665)
Supplement: gkaa665_Supplemental_File [file gkaa665_supplemental_file.pdf]

Atypical structures of GAA/TTC trinucleotide repeats underlying Friedreich's Ataxia: DNA triplexes and RNA/DNA hybrids: Supporting Information

Jiahui Zhang, Ashkan Fakharzadeh, Feng Pan, Christopher Roland, and Celeste Sagui*

Department of Physics, North Carolina State University, Raleigh, NC 27695-8202, USA

E-mail: sagui@ncsu.edu

1. Initial structures and statistical structural analysis of the molecular dynamics trajectories

Figure S1: Schematic illustrating the initial construction of the triple helices.

Figure S2: Cartoon illustrating the calculation of the effective number of hydrogen bonds and the effective stacking area for the third strand. Shown are: (a) a configuration illustrating that simply counting hydrogen bonds is not a good measure for structural stability; (b) extreme example of configuration where all bases are stacked but there is no overlap; and (c) details of how to calculate the effective number of hydrogen bonds and stacking area. Note that hydrogen bonds here are indicated by dashed red lines, bases by blue and yellow squares, and normals by blue arrows. Please see text for a more detailed discussion.

2. Coexisting Hydrogen Bond (HB) Conformations of Select Middle Steps in Triplexes

Figure S3: Coexisting HBs for stable antiparallel TTC·GAA:TTC DNA triplexes (there are no changes from the initial guess for the stable parallel TTC·GAA:TTC DNA triplexes).

Figure S4: Coexisting HBs for stable parallel GAA·GAA:TTC DNA triplexes.

Figure S5: Coexisting HBs for stable antiparallel GAA·GAA:TTC DNA triplexes.

Figure S13: Coexisting HBs for stable parallel d(GA$^+$A)·r(GAA):d(TTC) (shifted) hybrid triplexes.

Figure S14: Dominant HBs for stable parallel d(GAA)·r(GAA):d(TTC) and d(GA$^+$A)·r(GAA):d(TTC) (shifted) hybrid triplexes.

3. Non-protonated pY(a) and apY(a)

Figure S6: Final structure and the hydrogen bond and stacking analysis of non-protonated apY(a).

Figure S7: Final structure; hydrogen bond and stacking analysis; stable hydrogen bond of middle steps; and coexisting hydrogen bond patterns of non-protonated pY(a).

4. Higher temperature MD simulations and Ion Distributions

Figure S8: Time evolution of the RMSD of the most stable triplexes as selected by the conformational analysis for the 1 μs simulations at 300K. Yellow, orange and red boxes correspond to temperatures at 320K, 340K, 360K, respectively.

Figure S9: The final configuration of the apR(a) simulation in the presence of 0.2M Mg^{2+} ions (upper left panel), the effective stacking area versus effective hydrogen bond number of apR(a) under 0.2M Mg^{2+} (upper right panel), the corresponding hydrogen bond patterns for the middle a base planes (lower left panel) and the coexisting hydrogen bond pattern for the middle step (lower right panel).

Figure S10: Ion cloud densities around the pY, pR and hybrid pR triplexes. The cyan surfaces denote regions of higher ion density and the grey areas denote regions of lower ion density.

5. Structural Characterization of Hybrid Duplexes and Triplexes

To calculate the basepair and step parameters of double helices (either free-standing helices or belonging to a triplex) we used the 3DNA software package.[1,2] The regular z-axis defined in 3DNA was used, which is the average of two base normals, taking into consideration the M-N vs M+N basepair classification. The RMSD in Fig. S12 was computed with the cpptraj software,[3] where the reference set of coordinates are from the 800th frame (ns).

Figure S11: Examples of average basepair inclination and basepair-step twist, roll and helical rise for the DNA duplexes that form part of the pY and pR(+)-S triplexes.

Figure S12: RMSD as function of time (ns) (left) and RMSD distribution (right) for the four different hybrid double helices.

Figure S15: Average basepair inclination and basepair-step twist, roll and helical rise for the hybrid duplexes r(GAA):d(TTC) either as free-standing duplexes or as part of the DNA·RNA:DNA triplex.

6. Other

Figure S16: Cartoon illustrating the possible formation of parallel DNA R·R:Y triplexes.

References

(1) Lu, X. J.; Olson, W. K. 3DNA: A software package for the analysis, rebuilding and visualization of three-dimensional nucleic acid structures. *Nucleic Acids Research* **2003**, *31*, 5108 – 5121.

(2) Colasanti, A. V.; Lu, X.-J.; Olson, W. K. Analyzing and building nucleic acid structures with 3DNA. *Journal of visualized experiments: JoVE* **2013**,

(3) Roe, D. R.; Cheatham, T. E. PTRAJ and CPPTRAJ: Software for Processing and Analysis of Molecular Dynamics Trajectory Data. *J. Chem. Theory Comput.* **2013**, *9*, 3084–3095.

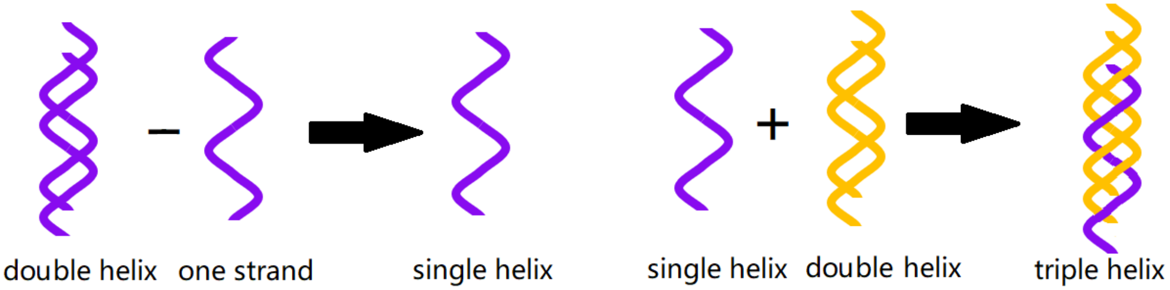

Figure S1: Schematic illustrating the initial construction of the triple helices.

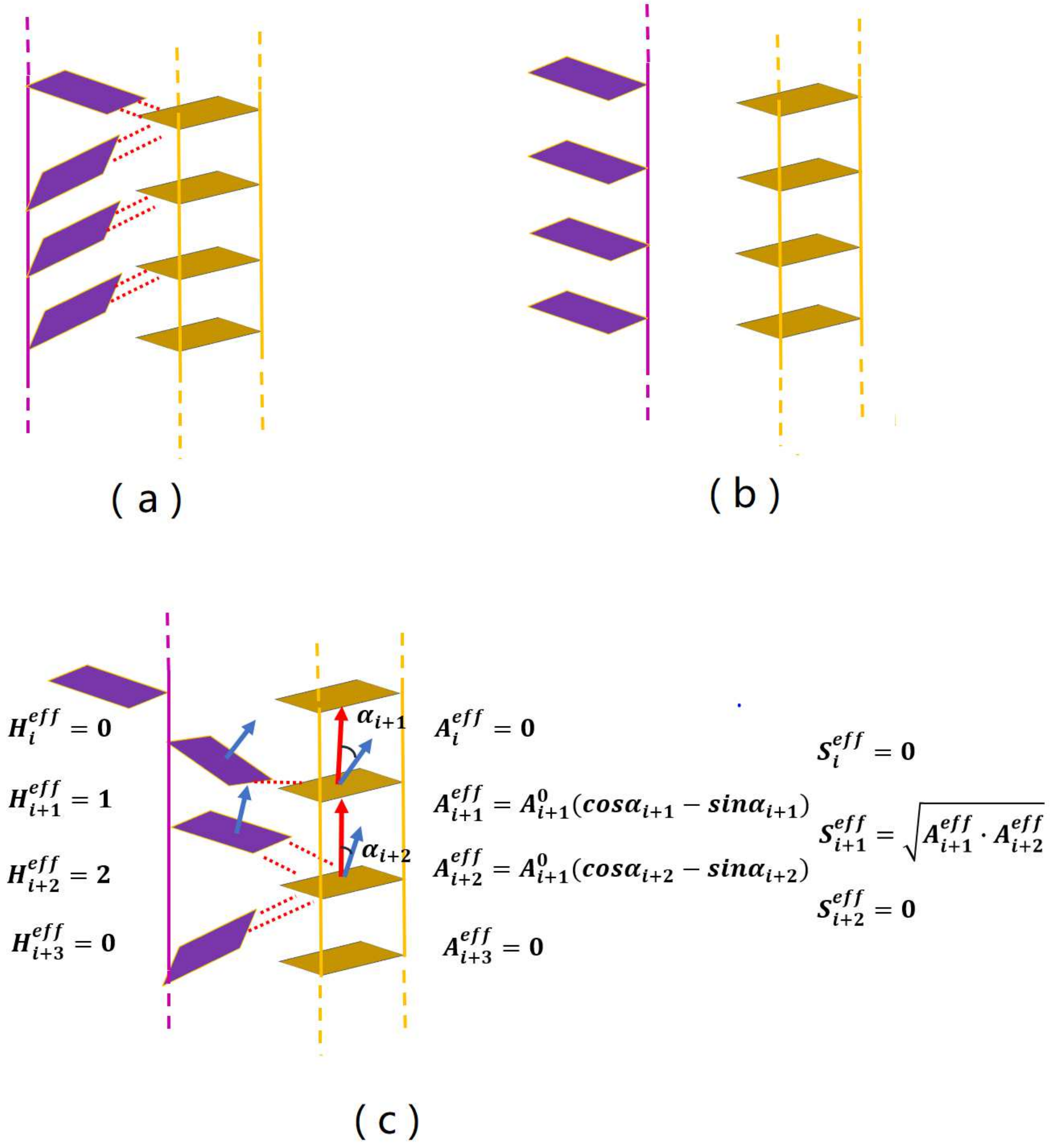

Figure S2: Cartoon illustrating the calculation of the effective number of hydrogen bonds and the effective stacking area for the third strand. Shown are: (a) a configuration illustrating that simply counting hydrogen bonds is not a good measure for structural stability; (b) extreme example of configuration where all bases are stacked but there is no overlap; and (c) details of how to calculate the effective number of hydrogen bonds and stacking area. Note that hydrogen bonds here are indicated by dashed red lines, bases by blue and yellow squares, and normals by blue arrows. Please see text for a more detailed discussion.

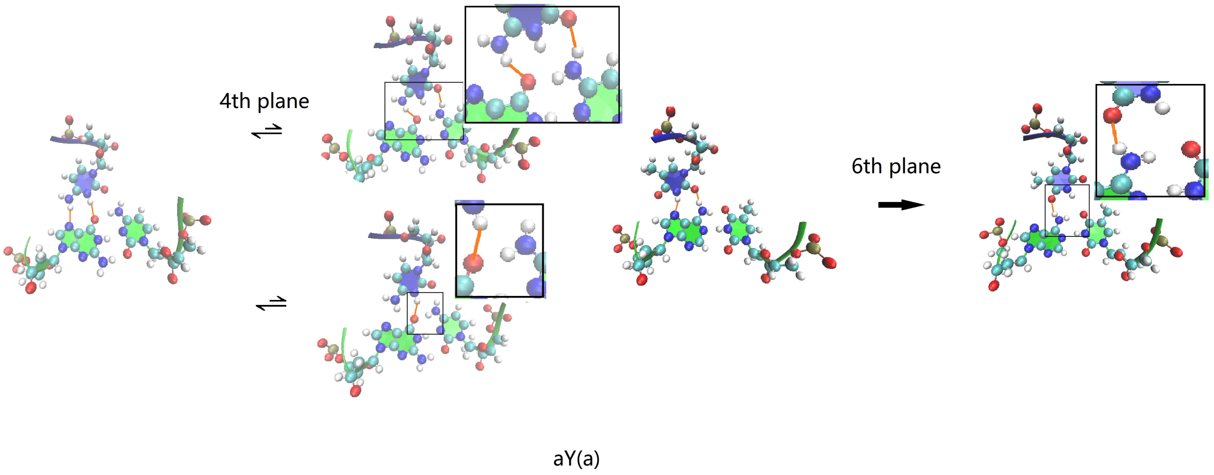

Figure S3: Coexisting hydrogen bonds for the middle steps of the stable Y·R:Y triplexes.

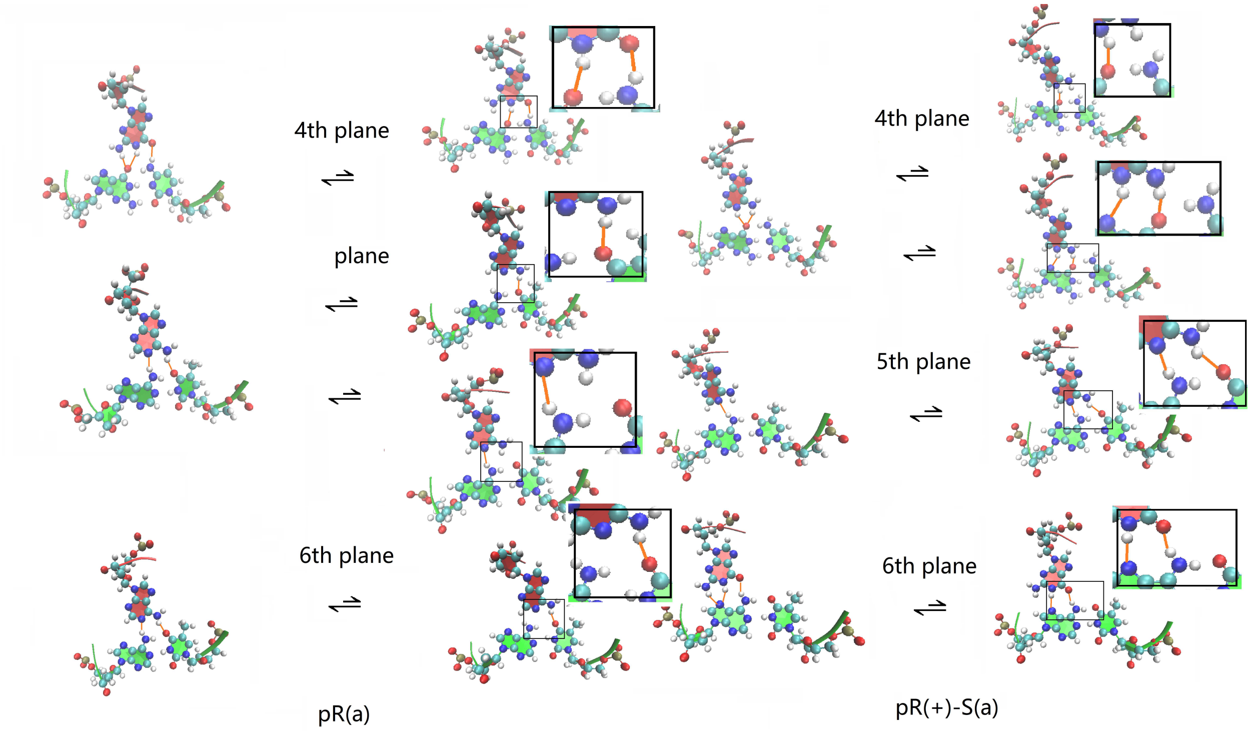

Figure S4: Coexisting hydrogen bonds for the middle steps of the stable parallel R·R:Y triplexes.

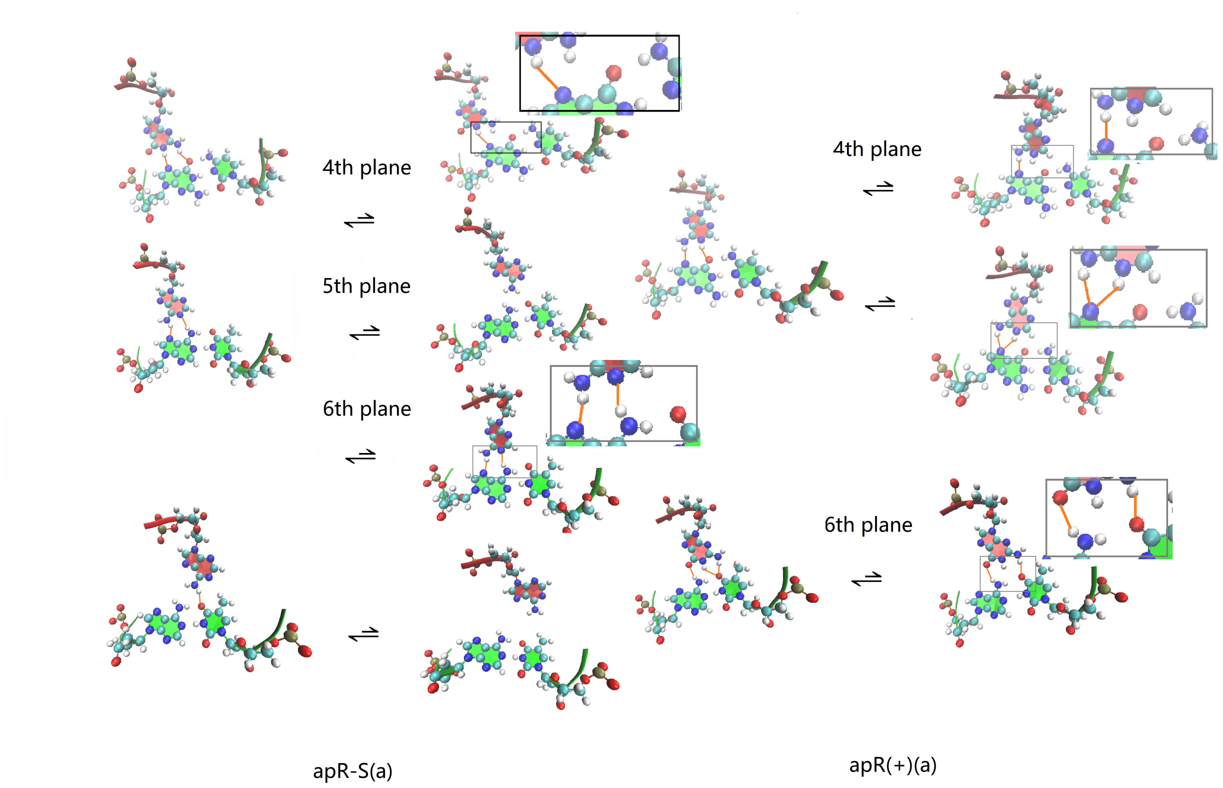

Figure S5: Coexisting hydrogen bonds for the middle steps of the stable antiparallel R·R:Y triplexes.

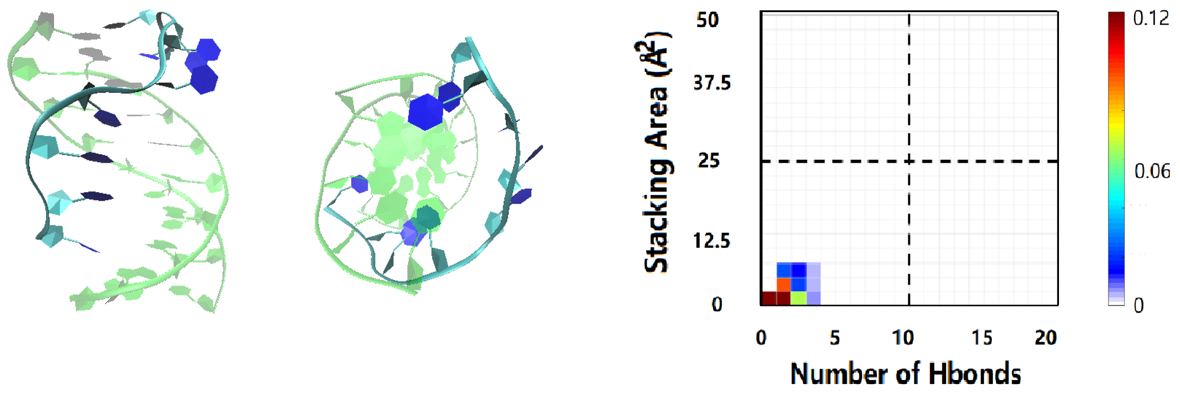

Figure S6: The final MD structure of the non-protonated apY(a) (left panel) and the corresponding stability diagram based on the effective stacking area and hydrogen bond number (right panel).

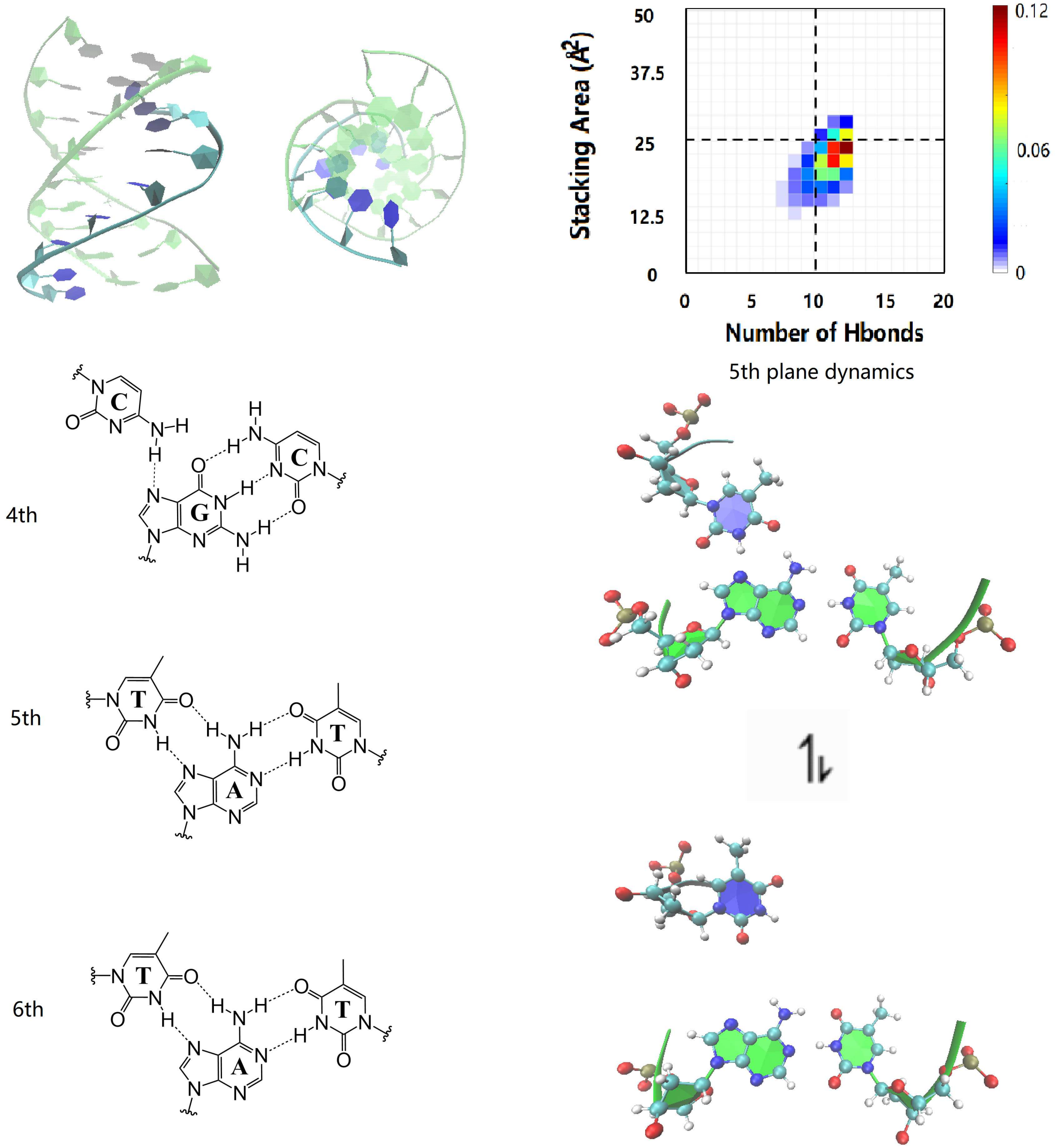

Figure S7: The final structure of the non-protonated pY(a) (upper left panel), the corresponding stability diagram based on the effective stacking area versus hydrogen bond number (upper right panel), the dominant hydrogen bond patterns for the middle base planes of non-protonated pY(a) (lower left panels) and the coexisting hydrogen bond pattern for the middle step for non-protonated pY(a)(lower right panels).

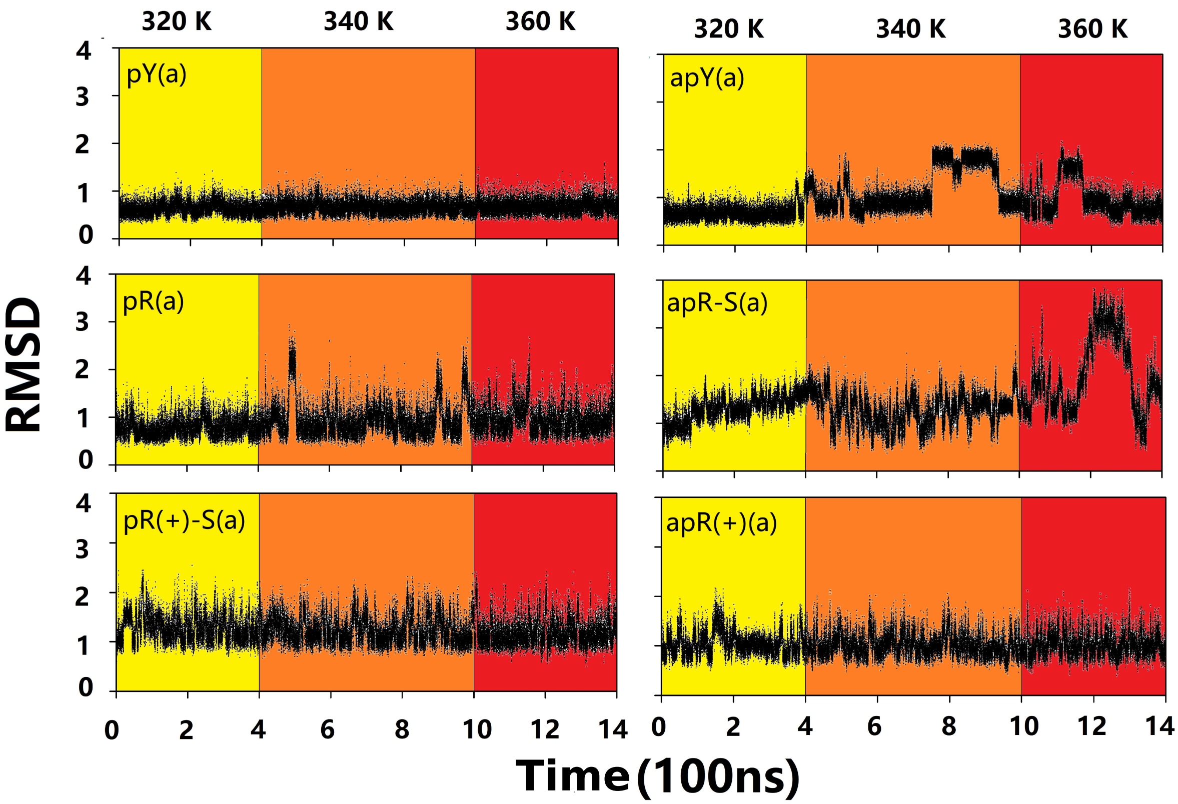

Figure S8: The time evolution of the RMSD of the most stable triplexes as selected by the conformational analysis for the 1 μs simulations at 300K. Yellow, orange and red boxes correspond to 320K, 340K and 380K, respectively.

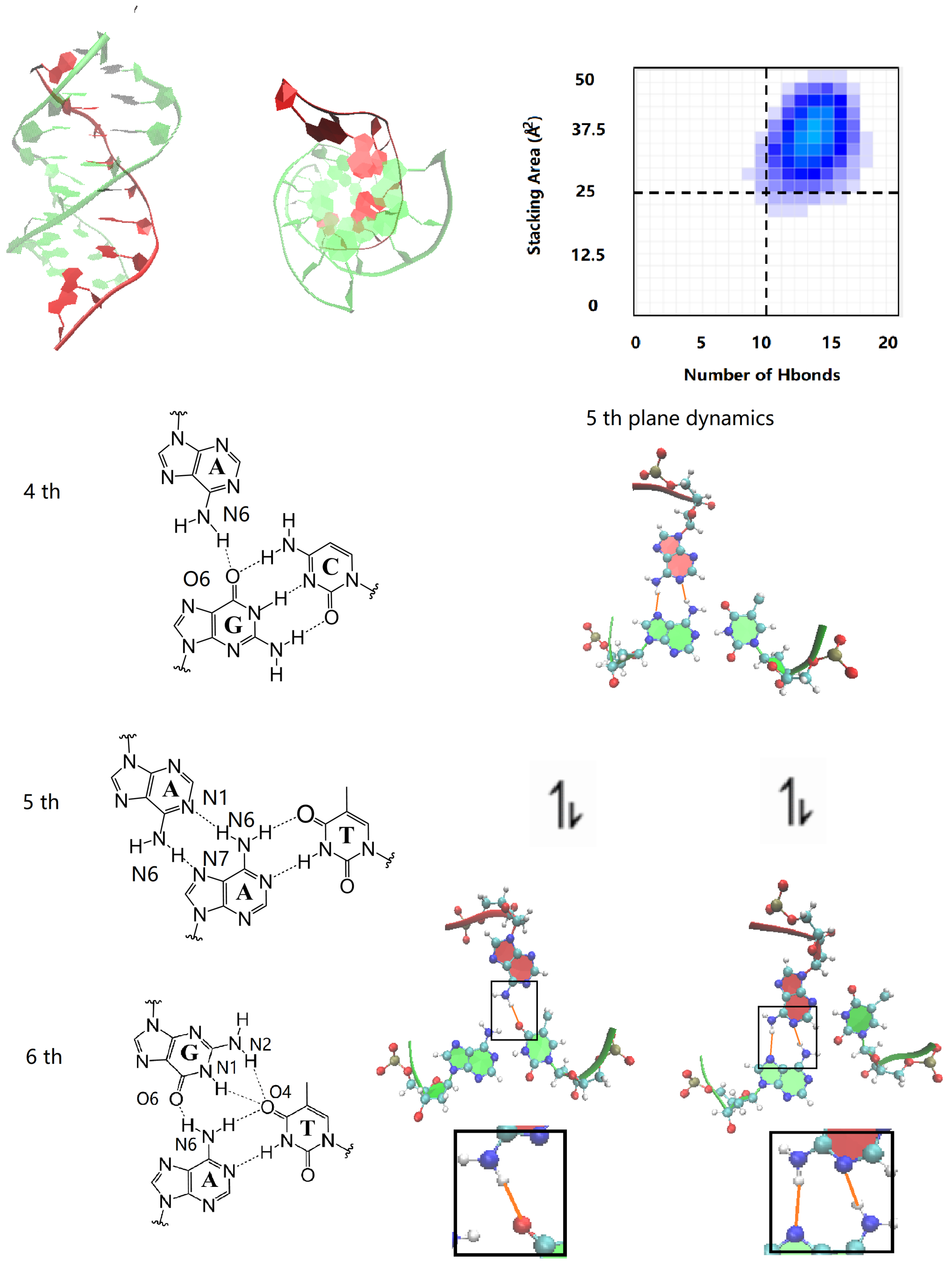

Figure S9: The final configuration of the apR(a) simulation in the presence of 0.2M Mg^{2+} ions (left upper panel), the effective stacking area versus effective hydrogen bond number of apR(a) under 0.2M Mg^{2+} (right upper panel), the corresponding hydrogen bond patterns for the middle base planes (left lower panel) and the coexisting hydrogen bond pattern for the middle step (right lower panel).

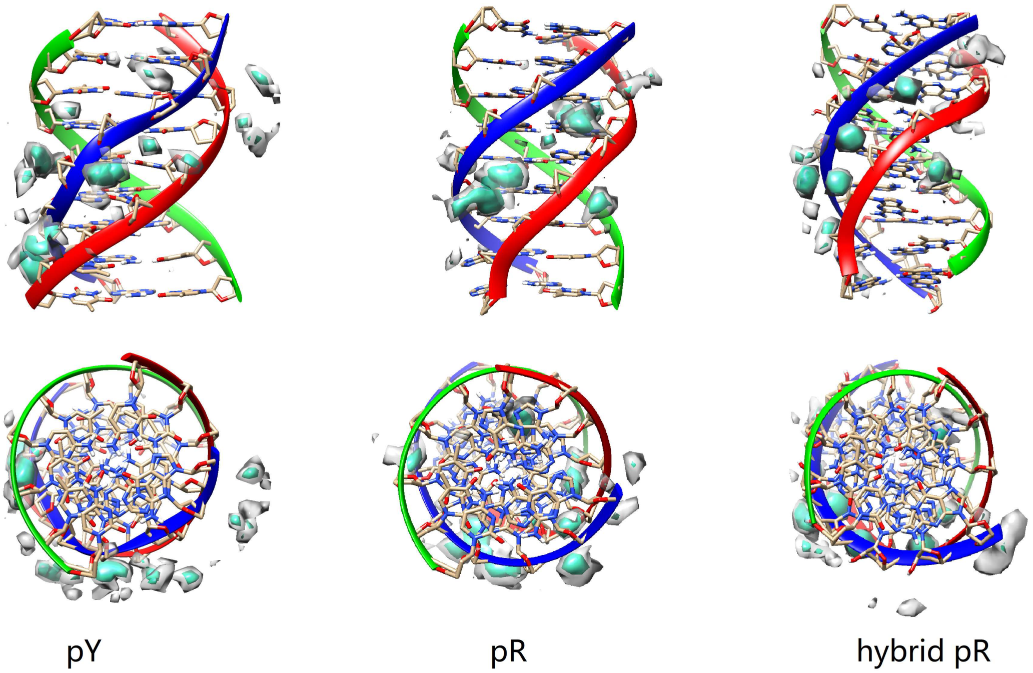

Figure S10: Ion cloud densities around the pY, pR and hybrid pR triplexes. The cyan surfaces denote regions of higher ion density, and the grey surfaces denote regions of lower ion density. Colors as follows: Red, third strand; blue, GAA strand of the duplex; green, TTC strand of the duplex.

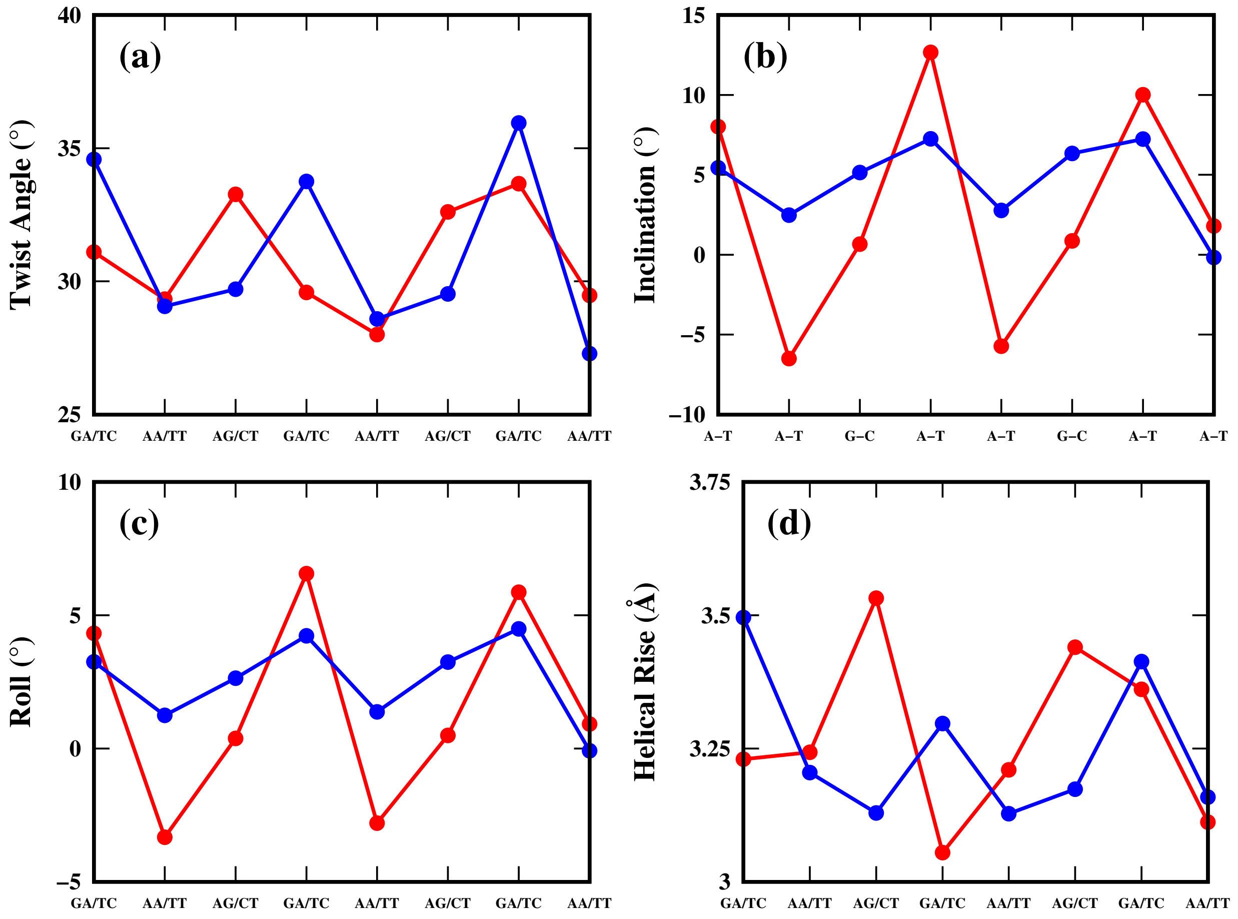

Figure S11: Average basepair inclination and basepair-step twist, roll and helical rise for the DNA duplexes that form part of the pY (blue) and pR(+)-S (red) triplexes. Data was averaged over the last 200ns.

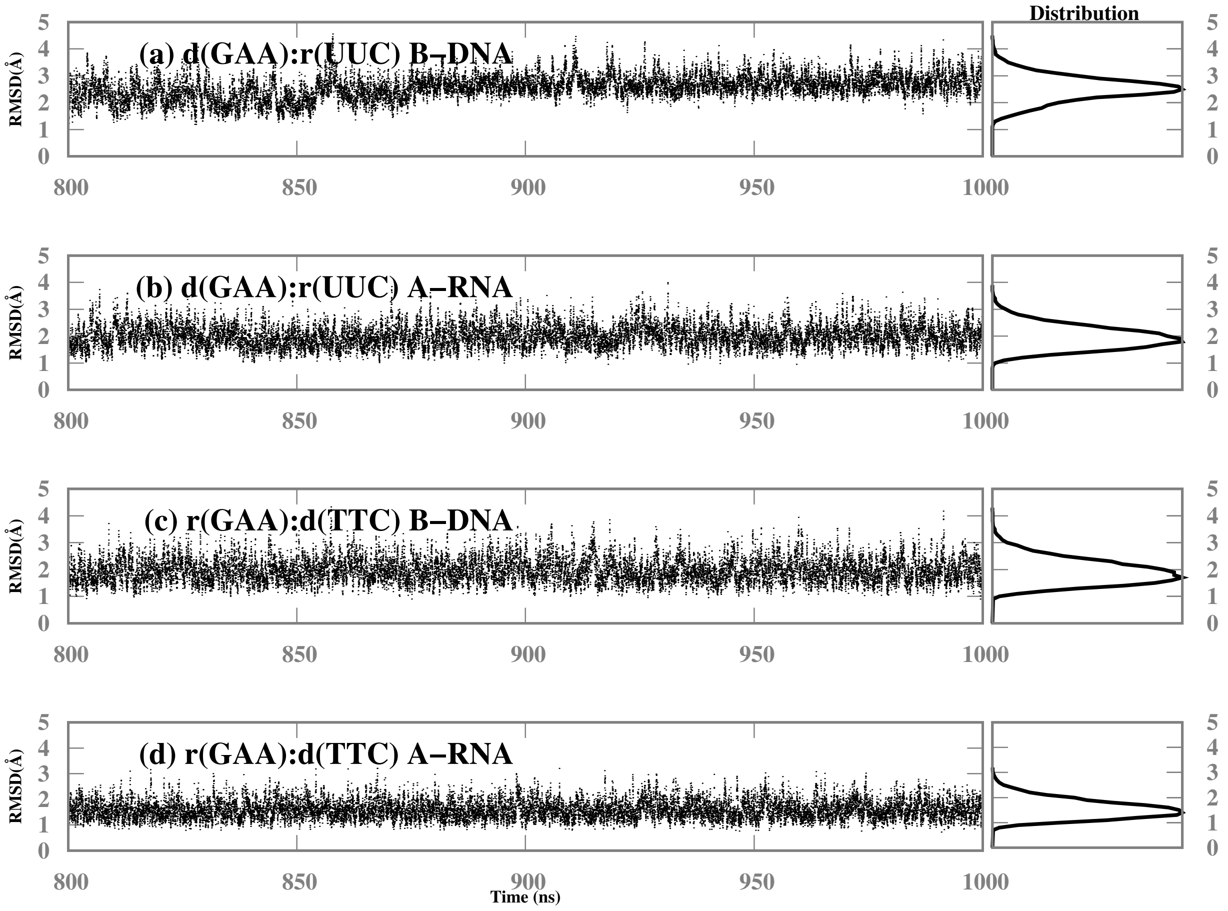

Figure S12: RMSD as function of time (ns) with respect to the 800th frame (left) and RMSD distribution (right) for the four different hybrid double helices.

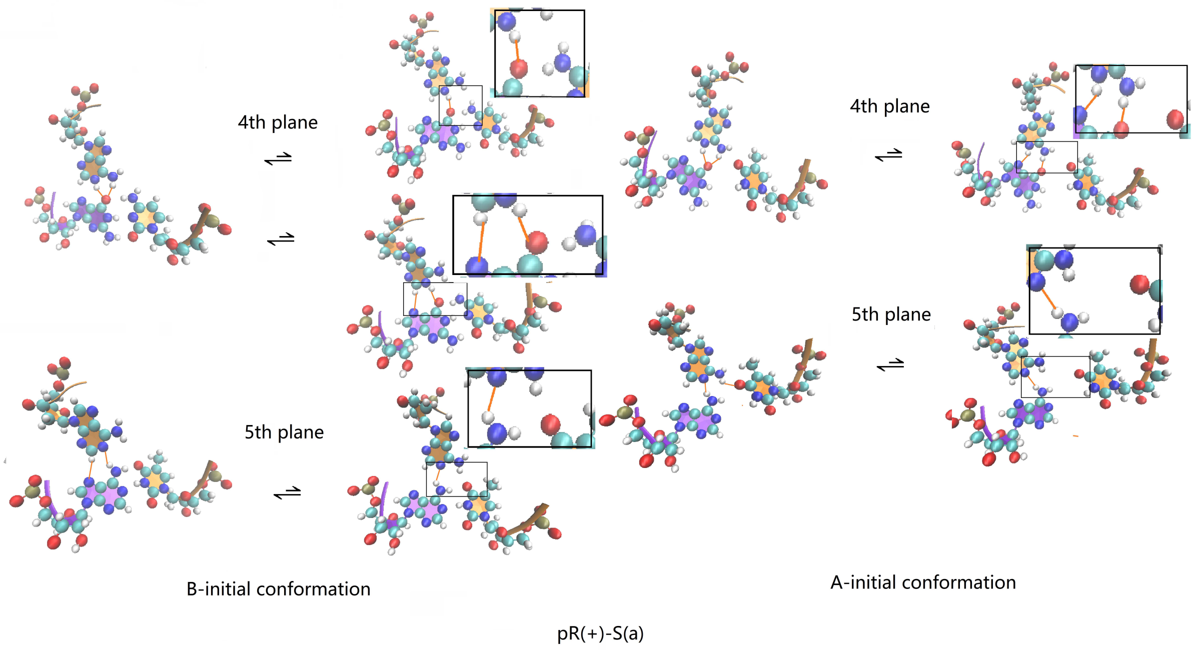

Figure S13: Coexisting hydrogen bonds for the middle steps of the pR(+)-S hybrid triplex.

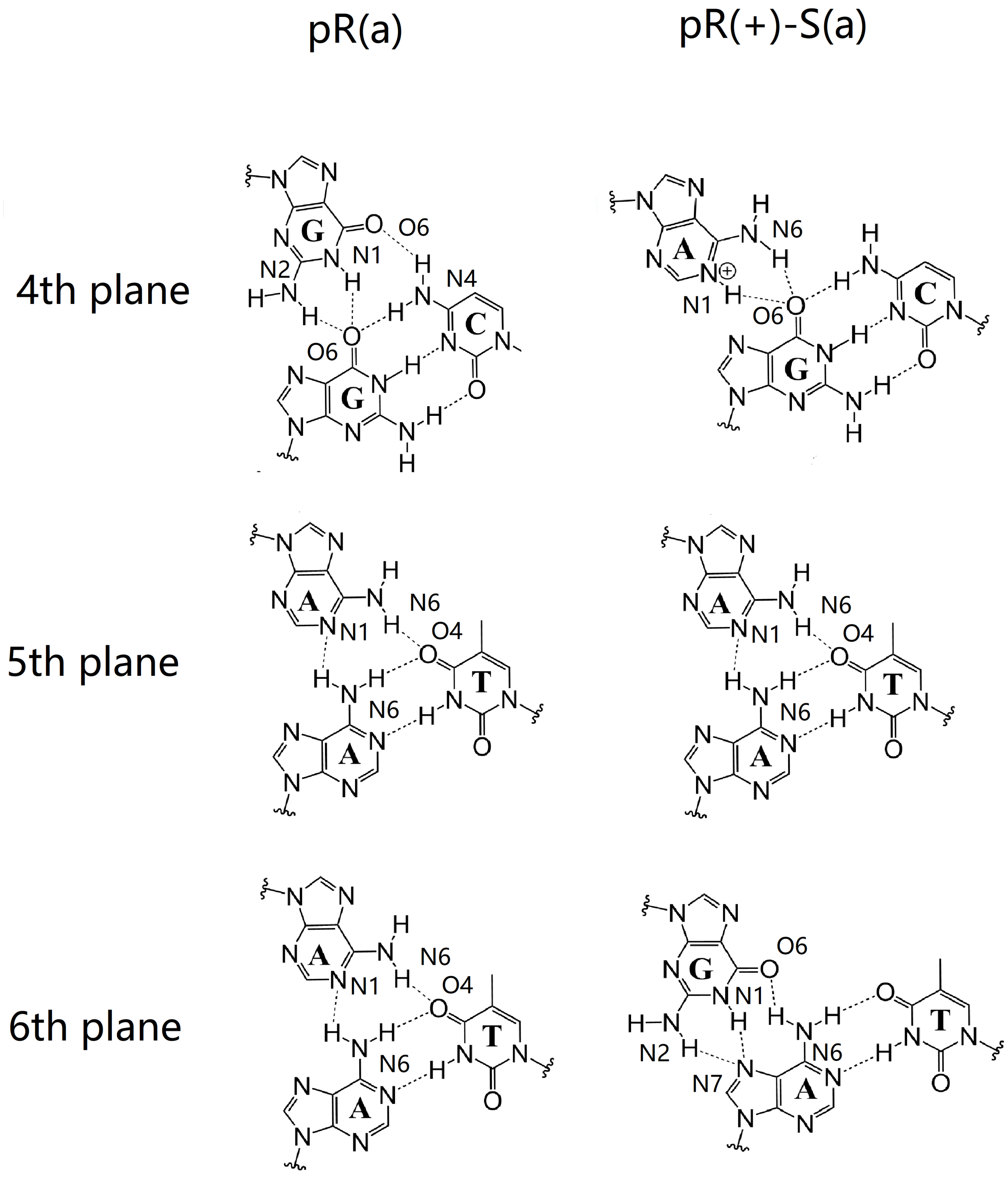

Figure S14: Dominant hydrogen bond patterns for the middle base planes of the stable hybrid DNA/RNA triplexes.

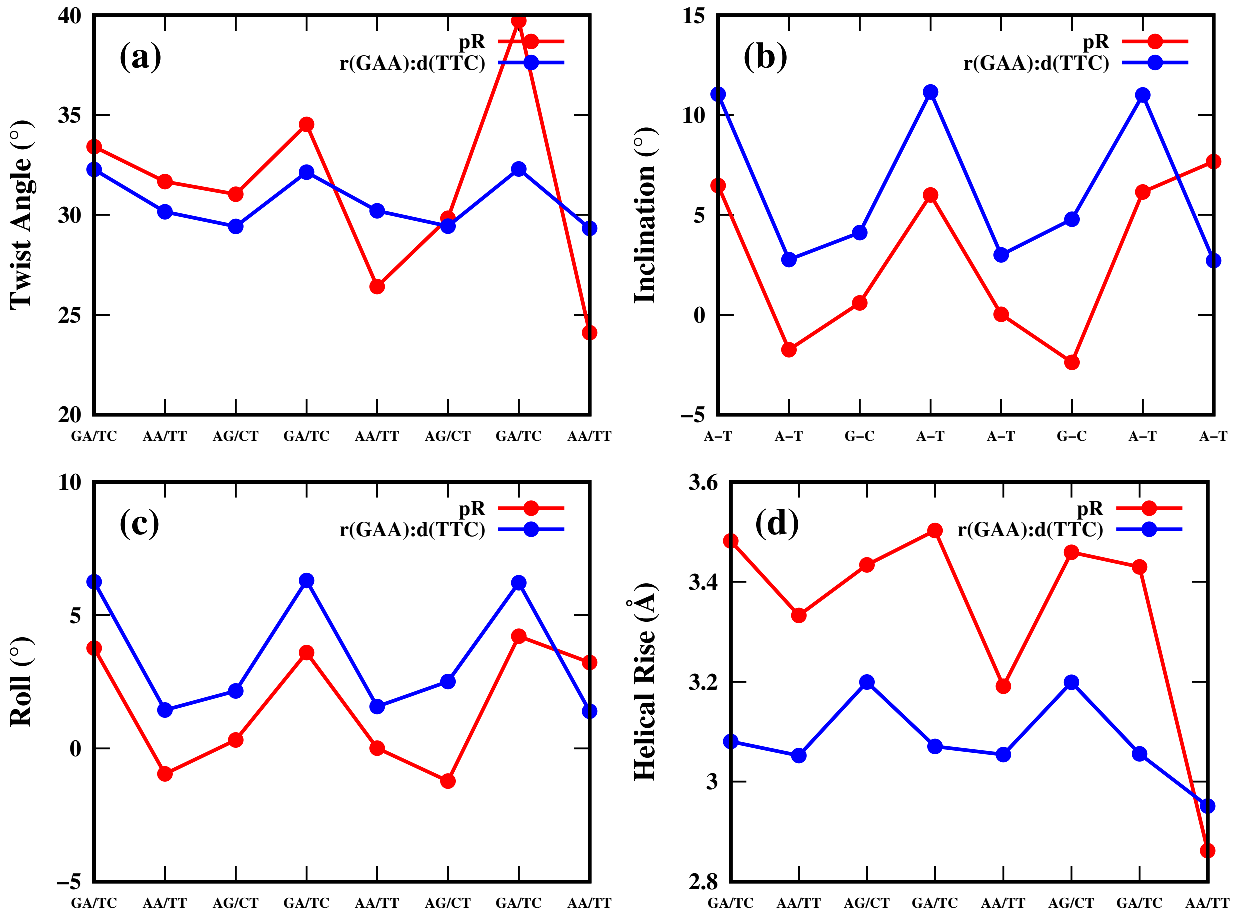

Figure S15: Average basepair inclination and basepair-step twist, roll and helical rise for the hybrid duplexes r(GAA):d(TTC) either as free-standing duplexes (blue) or as part of the DNA·RNA:DNA triplex (red). Data was averaged over the last 200ns.

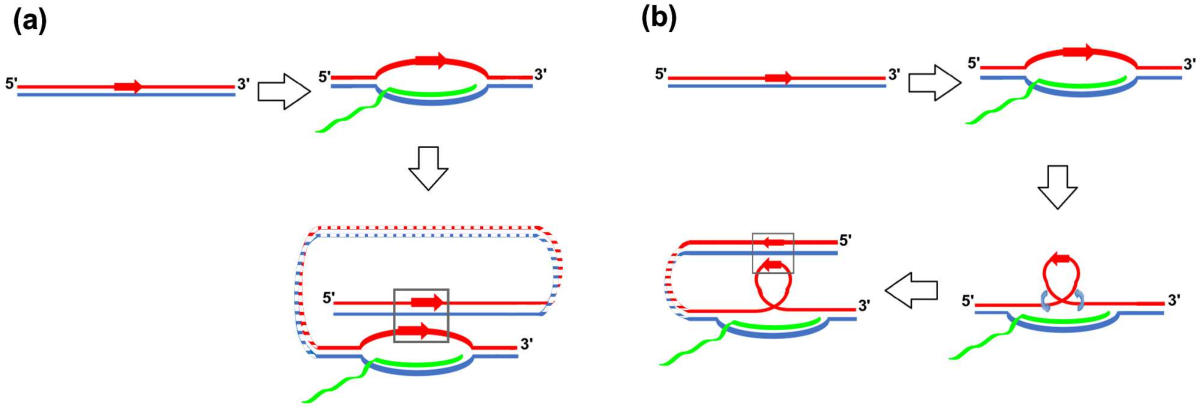

Figure S16: Cartoons illustrating possible pathways for the formation of parallel DNA GAA·GAA:TTC triplexes. Red: GAA DNA coding strand; Blue: TTC DNA template strand; Green: mRNA. Once the two strands are separated in an R-loop, the GAA strand can form a parallel triplex with a segment of duplex DNA either intermolecularly (omitting dotted lines in cartoon, *e.g.* in plasmids) or intramolecularly (with dotted lines in cartoon). In the intermolecular case, a segment of a second duplex approaches the GAA strand with the right orientation in (a) or (b). In the intramolecular case, a duplex turns more than once (a) to approach the GAA single strand. In (b), negative supercoiling causes a rotation at the base of the GAA sstrand, and a single turn of the duplex is enough to bring it in parallel alignment with the GAA sstrand.
